# Developmental excitatory-to-inhibitory GABA-polarity switch is disrupted in 22q11.2 deletion syndrome: a potential target for clinical therapeutics

**DOI:** 10.1038/s41598-017-15793-9

**Published:** 2017-11-16

**Authors:** Hayder Amin, Federica Marinaro, Davide De Pietri Tonelli, Luca Berdondini

**Affiliations:** 1 Nets3 Laboratory, Department of Neuroscience and Brain Technologies (NBT), Fondazione Istituto Italiano di Tecnologia (IIT), Via Morego 30, 16163 Genoa, Italy; 2 Neurobiology of miRNA Laboratory, Department of Neuroscience and Brain Technologies (NBT), Fondazione Istituto Italiano di Tecnologia (IIT), Via Morego 30, 16163 Genoa, Italy; 3 Present Address: Gurdon Institute and Dept. of Biochemistry, University of Cambridge, Tennis court road, Cambridge, CB2 1QN UK

## Abstract

Individuals with 22q11.2 microdeletion syndrome (22q11.2 DS) show cognitive and behavioral dysfunctions, developmental delays in childhood and risk of developing schizophrenia and autism. Despite extensive previous studies in adult animal models, a possible embryonic root of this syndrome has not been determined. Here, in neurons from a 22q11.2 DS mouse model (*Lgdel* ^+/−^), we found embryonic-premature alterations in the neuronal chloride cotransporters indicated by dysregulated NKCC1 and KCC2 protein expression levels. We demonstrate with large-scale spiking activity recordings a concurrent deregulation of the spontaneous network activity and homeostatic network plasticity. Additionally, *Lgdel* ^+/−^ networks at early development show abnormal neuritogenesis and void of synchronized spontaneous activity. Furthermore, parallel experiments on *Dgcr8* ^+/−^ mouse cultures reveal a significant, yet not exclusive contribution of the *dgcr8* gene to our phenotypes of *Lgdel* ^+/−^ networks. Finally, we show that application of bumetanide, an inhibitor of NKCC1, significantly decreases the hyper-excitable action of GABA_A_ receptor signaling and restores network homeostatic plasticity in *Lgdel* ^+/−^ networks. Overall, by exploiting an on-a-chip 22q11.2 DS model, our results suggest a delayed GABA-switch in *Lgdel* ^+/−^ neurons, which may contribute to a delayed embryonic development. Prospectively, acting on the GABA-polarity switch offers a potential target for 22q11.2 DS therapeutic intervention.

## Introduction

The 22q11.2 deletion syndrome (22q11.2 DS), also known as DiGeorge Syndrome (DGS), confers the most significant genetic risk for developing schizophrenia (25–30%) in early adulthood^1^, autism spectrum disorders (20–25%)^2^ and predisposition to different psychiatric disorders including cognitive, learning, and behavioral impairments (>60%)^3,4^. The syndrome occurs in 1 of every 4000 live-births^5^ and caused by hemizygous deletion of 1.5–3 megabases (Mb) within the region q11.2 of the human chromosome 22^4,6^.

Neuropathology studies have suggested that neurodevelopmental abnormalities occurring in the early brain formation may play a vital role in the pathogenesis of the complex neuropsychiatric phenotypes of this syndrome. Additionally, a wealth of data has demonstrated that the distribution and function of gamma-aminobutyric acid (GABA)-ergic-interneurons are perturbed in the brains of adult 22q11.2 DS patients^7^ and that impaired GABAergic-signaling is involved in several neurodevelopmental disorders such as fragile×^8^, autism^9^, schizophrenia^10^, Rett Syndrome^9^, and Down Syndrome^11^. GABA is the primary inhibitory neurotransmitter in the adult central nervous system (CNS), but in fetal life and early postnatal development, it acts mostly as excitatory, exerting a vital trophic and regulatory role for the maturation of neurons^12^. Alterations in the GABA-polarity switch can cause a delay in the functional maturation of developing neural circuits^13^, consequences for network excitability^14^ and can give rise to neuropsychiatric phenotypes. However, impairments associated with the GABAergic-signaling and correlated network dysfunctions at the early onset of brain circuit development have not yet been reported in the 22q11.2 DS.

During early brain development, immature neurons have a high intracellular chloride concentration [Cl^−^]_int._, thus their GABA_A_ receptors (GABA_A_Rs)-mediated-signaling sets for depolarization and action potential generation, leading to activation of NMDA receptors (NMDARs) and hence inducing an elevation of Ca^2+^ concentration^15^. This excitatory-depolarizing property of GABA is mediated by developmental changes in the expression of neuronal cation-chloride cotransporters (CCCs) that regulate the neuronal chloride homeostasis^16^. Therefore, in embryonic and early postnatal life, the expression of accumulating-chloride Na^+^-K^+^-Cl^−^ cotransporter (NKCC1) is high, while on later developmental stages the expression of extruding-chloride K^+^-Cl^−^ cotransporter (KCC2) increases^17,18^. Thus, this dynamic change in the expression of NKCC1 to KCC2 sets an accurate developmental switch of GABAergic-signaling from excitatory-to-inhibitory.

The switch of GABAergic-signaling is one of the drivers that modify the spontaneous activity in early neuronal development. This spontaneous activity plays a fundamental role during critical stages of early brain circuits’ development and in their orchestrated activity-dependent formation^19,20^. On the other hand, several lines of evidence indicate the essential role of neuronal electrical activity to regulate the synaptic strength and to refine the maturation of developing neural circuits within a neuronal network^20^. Additionally, in very early brain development, the abnormal neuronal activity can alter the critical homeostatic balance between excitatory (Glutamatergic) and inhibitory (GABAergic) neurotransmission and may contribute to many neurological disorders such as autism, schizophrenia, and epilepsy^21,22^. GABAergic synapses and receptors are initiated before Glutamatergic ones in the hippocampus and other regions of the brain^23^; hence, GABAergic-signaling plays an important role for processing and storage of information by controlling signaling cascades that regulate maturation, survival, and differentiation and promote synaptic stabilization and connection, and neuronal network wiring^19^. Interestingly, the fraction of GABAergic-neurons in rats is already established during the embryonic period from E10.5 through E18.5 and several days after birth^24^, and the activity oscillation of these interneurons sets the melody for the early network activity^25^. In turn, this evidence suggests that altered GABA-ergic signaling might be a key factor in the pathophysiology of neural deficits observed in 22q11.2 DS. Several studies performed in adult brains have provided compelling evidence on the neuronal impairments and brain dysfunctions associated with the 22q11.2 microdeletion, but the pathogenesis at early onset of brain development has remained to be investigated. To access the molecular, cellular, synaptic, and circuit-level changes underlying the accurate human phenotype of this syndrome, several mouse models were designed to carry chromosomal defects, which are syntenic to the human 22q11.2 deletion. Two of these multigene deletion models, *Df*(16*A* ^+/−^)^26^ and *Lgdel* ^+/−^^27^, carry a hemizygous deletion in the mouse chromosome 16 that is syntenic to the 1.5 Mb microdeletion of the human chromosome 22q11.2. Notably, these mouse models have become standard models for studying schizophrenia due to their common facets with the 22q11 deletion in schizophrenia. In addition, mouse models carrying single-gene mutations were also generated, including a heterozygous mouse model for the *Dgcr8* ^+/−^^28^, which carries a deletion in the DGS chromosomal region 8 (*dgcr8*) gene, one of the deleted genes of the 22q11.2 microdeletion. This gene is a key component of the Microprocessor complex required for biogenesis of microRNA (miRNA)^29^ and holds a fundamental role in the molecular control of brain development^30^. Effects of the *dgcr8* gene deletion were directly associated with the pathophysiology and cognitive phenotypes of the 22q11.2 DS^26,31^.

**Figure 1:**
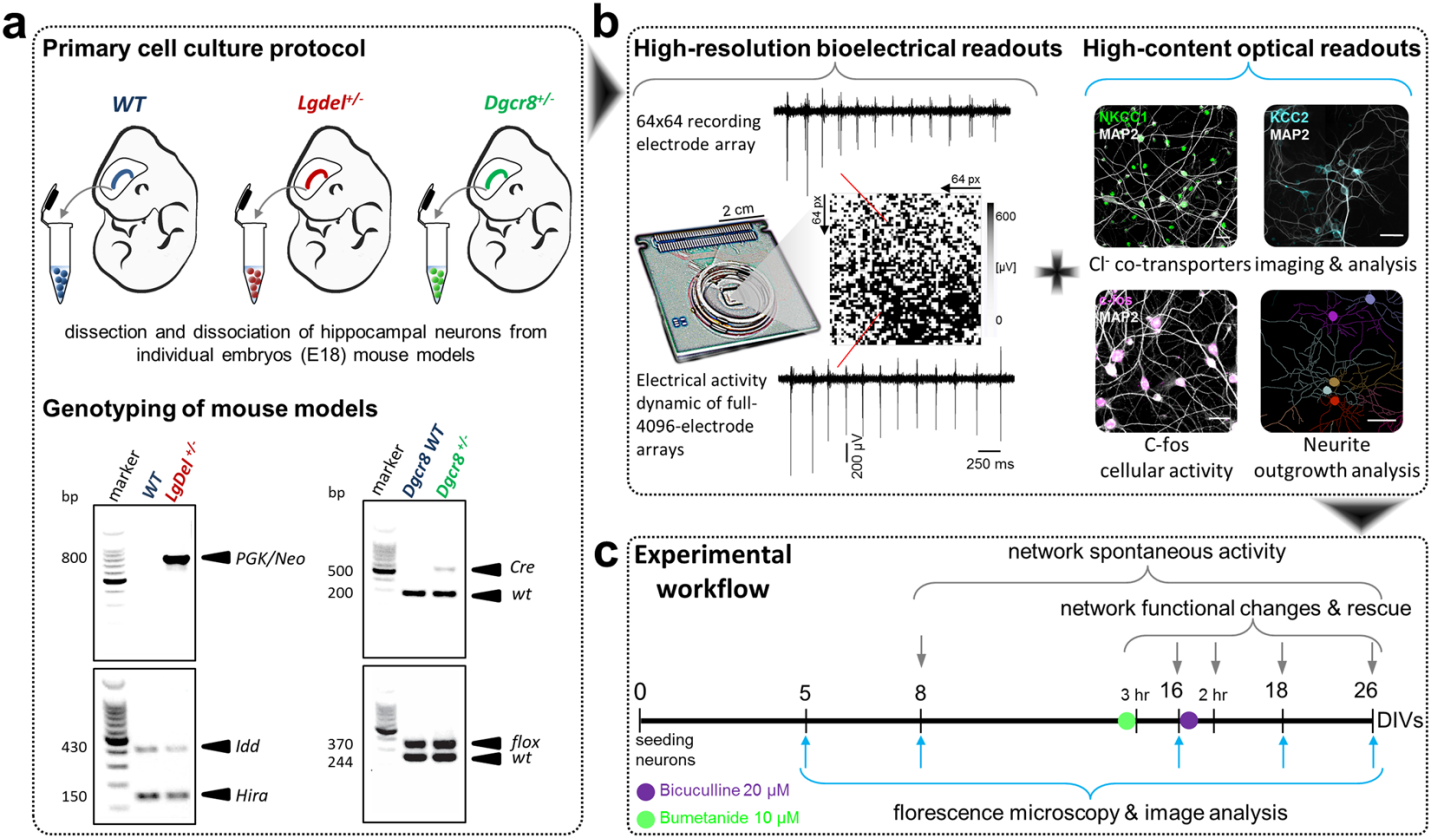
Schematic of experimental implementation of the 22q11.2 DS model. (**a**) Single-embryo primary cell culture preparation of hippocampal neurons from WT, *Lgdel* ^+/−^ and *Dgcr8* ^+/−^ mice and their genotyping. Each cropped gel image reports two representative embryos from groups of animals genotyped for *PGK*/*Neo* (*top left*) and *Idd*/*Hira* alleles (*bottom left*) in (WT and *Lgdel* ^+/−^ embryos) or *Cre* (*top right*) and *flox* alleles (*bottom right*) in (Dgcr8 WT and *Dgcr8* ^+/−^ embryos); “full-length gels are presented in Supplementary Figure S5”. (**b**) Overview of the experimental readouts used in this study, combining electrical measures and optical HCI. Scale bars represent 30 μm. (**c**) Workflow timeline of the experimental implementation.

Here, we investigated the role of the switch of GABAergic-signaling in developing neuronal networks prepared from *Lgdel* ^+/−^ and *Dgcr8* ^+/−^ mouse embryos (Fig. 1a). Specifically, we performed fluorescence high-content imaging (HCI) microscopy and high-resolution bioelectrical extracellular signal recordings using 4096-electrodes complementary metal-oxide semiconductor-multielectrode arrays (CMOS-MEAs) on cultured networks (Fig. 1b,c). Notably, the GABA-polarity switch has been reported to occur at ~10 days-*in vitro* (DIVs)^18^ in embryonic rat hippocampal neuronal cultures, and within the second postnatal week ~P9^32^ in rat hippocampal slices. Here, hippocampal neurons from embryonic wild-type (WT), *Lgdel* ^+/−^ and *Dgcr8* ^+/−^ mice (Fig. 1a and Supplementary Figure S5) were plated at a time when GABA was excitatory (E18.5, 2.5 days before birth)^18^. The WT refers to the phenotype of the typical form of a mouse as it occurs in nature. Therefore, the WT has a normal genotype, in contrast to the mutant genotypes in *Lgdel* ^+/−^ and *Dgcr8* ^+/−^ mice; hence, it is considered the control mouse model in our experiments. The expression of NKCC1 and KCC2 as well as the spontaneous electrical activity were monitored and quantified over 26 DIVs. We found that diminished 22q11.2 gene dosage in an embryonic 22q11.2 DS *Lgdel* ^+/−^ mouse culture exhibited a noticeable delay in the regular excitatory-to-inhibitory switch of GABA-signaling during *in vitro* development, and showed abnormally high levels of NKCC1 even after three weeks in culture. Correlated with this delayed GABA-polarity switch observed in *Lgdel* ^+/−^, we found an altered spontaneous network activity, lack of synchronization and widespread homeostatic plasticity defects. Concurrent experiments performed on *Dgcr8* ^+/−^ mouse cultures allowed us to evaluate the contribution of *dgcr8* single-gene deletion to the GABA-polarity switch and early network dysfunctions on the multigene deletion of neuronal networks from *Lgdel* ^+/−^ mouse. Finally, the treatment of *Lgdel* ^+/−^ cultures with NKCC1 inhibitor bumetanide rescued the hyper-excitability of GABA-signaling and restored the homeostatic plasticity to levels seen in WT neuronal networks.

## Results

### Altered expression of NKCC1 and KCC2 in *Lgdel*^+/−^ and *Dgcr8*^+/−^ embryonic hippocampal neurons

**Figure 2:**
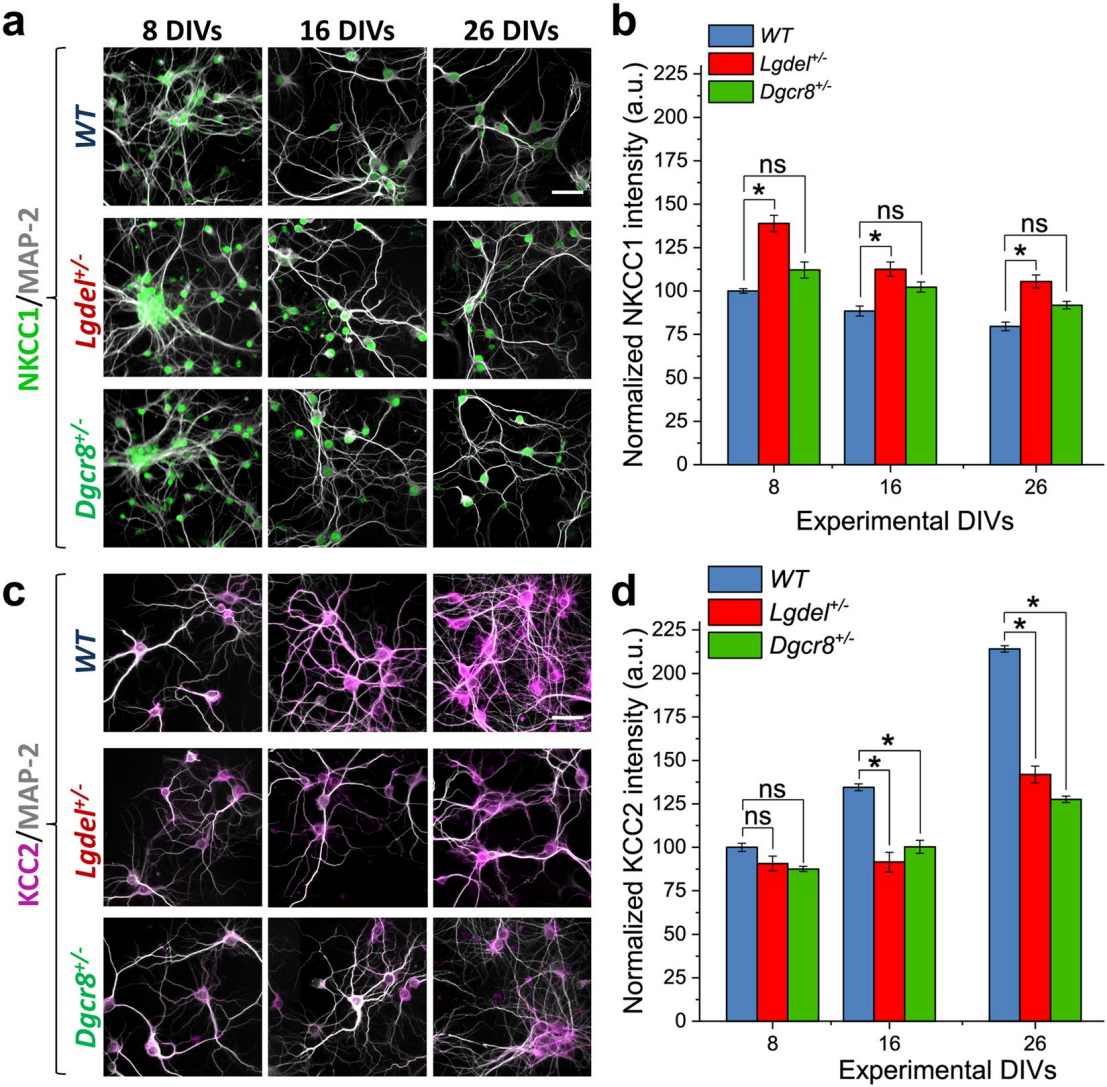
Altered expression of NKCC1 and KCC2 in *Lgdel* ^+/−^ and *Dgcr8* ^+/−^ neurons. (**a**) Fluorescence micrographs showing intensity expression of NKCC1 in cultures from WT, *Lgdel* ^+/−^ and *Dgcr8* ^+/−^ (n = 5), at 8, 16, 26 DIVs. Scale bar represents 30 μm. (**b**) Quantification of NKCC1 intensity shows a significantly higher expression in *Lgdel* ^+/−^ neurons (n = 5) compared to WT neurons (n = 7) across all developmental ages (*p < 0.05, ANOVA) and slightly higher than *Dgcr8* ^+/−^ neurons (n = 5) (ns denotes not significant). (**c**) Fluorescence micrographs showing the changes of intensity expression of KCC2 in cultures from the three animal genotypes at 8, 16, 26 DIVs. Scale bar represents 30 μm. (**d**) Quantification of KCC2 intensity at 8 DIVs shows the non-significant difference between genotypes (ns denotes not significant), but displaying a significantly lower expression in *Lgdel* ^+/−^ and *Dgcr8* ^+/−^ neurons compared to WT neurons at 16 and 26 DIVs (*p < 0.05, ANOVA).

To investigate whether the GABA-signaling was disrupted by the 22q11.2 microdeletion in *Lgdel* ^+/−^ mouse neuronal culture, we prepared primary hippocampal neurons from embryonic WT and *Lgdel* ^+/−^ mice that were grown in 96 wells imaging-microplates for up to 26 DIVs using optimized cell culture conditions (see Methods). In the same way, we also prepared cultures from *Dgcr8* ^+/−^ mouse embryos to characterize the effects of the *dgcr8* gene deletion. Then, by immunofluorescence and HCI, we measured the expression of the two chloride cotransporters NKCC1 and KCC2 at 8, 16, 26 DIVs. As shown in (Fig. 2a,b and Supplementary Figure S1a), the analysis of the fluorescence intensity for NKCC1 at 8 DIVs showed a significantly higher expression in *Lgdel* ^+/−^ (138.9 ± 4.67%; *p* < 0.05, ANOVA) compared to *Dgcr8* ^+/−^ (112.1 ± 4.67%) and to the WT samples. At 16 DIVs, NKCC1 significantly decreased in *Lgdel* ^+/−^ (112.5 ± 1.1%) compared to its expression at 8 DIVs, but it remained significantly higher than WT (88.4 ± 2.86%; *p* < 0.05, ANOVA) and slightly higher than *Dgcr8* ^+/−^ (102.3 ± 4.64%). Further, although at 26 DIVs the NKCC1 expression reached its lowest level in *Lgdel* ^+/−^ (105.5 ± 3.78%), its value remained significantly higher compared to *Dgcr8* ^+/−^ (91.8 ± 2.16%; *p* < 0.05, ANOVA) and WT (79.6 ± 2.5%; *p* < 0.05, ANOVA), respectively. On the other hand, as shown in (Fig. 2c,d and Supplementary Figure S1b), the analysis of the fluorescence intensity for KCC2 at 8 DIVs showed the lowest level across the developmental time points, with no significant differences for both *Lgdel* ^+/−^ (90.68 ± 2.23%) and *Dgcr8* ^+/−^ (87.6 ± 1.49%) compared to the WT samples. At 16 DIVs, KCC2 expression significantly increased in WT cultures (134.5 ± 1.96%) compared to its expression at 8 DIVs, which was also significantly higher compared to *Lgdel* ^+/−^ (91.5 ± 5.64%; *p* < 0.05, ANOVA) and *Dgcr8* ^+/−^ (100.29 ± 3.81%; *p* < 0.05, ANOVA) cultures, respectively. At 26 DIVs, WT cultures showed the highest level of KCC2 expression (214.13 ± 1.88%), while *Lgdel* ^+/−^ and *Dgcr8* ^+/−^cultures, albeit their increased KCC2 expression from 16 DIVs, displayed a significantly lower level of expression compared to WT (141.86 ± 4.82%; and 127.6 ± 1.78%; *p* < 0.05, ANOVA), respectively.

To assess whether the altered protein levels of NKCC1 and KCC2 were accompanied by changes in their transcript levels, we performed quantitative real-time polymerase chain reaction (qRT-PCR) on samples from WT, *Lgdel* ^+/−^ and *Dgcr8* ^+/−^ at the same time points as in previous experiments (8, 16 and 26 DIVS), by using specific primers for *NKCC1* and *KCC2* genes. We found a significant alteration of these transcripts only in *Lgdel* ^+/−^ cultures at 8 DIVs compared to WT cultures (Supplementary Figure S2a–d). Importantly, given that we quantified the cotransporters’ expression exclusively for neuronal populations, which is a void resolution in qRT-PCR readouts; this could account for the observed differences between protein and transcript levels in *Lgdel* ^+/−^ cultures compared to WT. On the other hand, the discordance between protein expression and transcripts may suggest a possible alteration in the post-transcriptional control of the cotransporters expression in *Lgdel* ^+/−^ cultures^33^.

Furthermore, the *dgcr8* gene (one of the deleted genes in the 22q11.2 microdeletion), is essential for the biogenesis of miRNAs. Thus, we also attempted to assess the miRNA expression profiles in cultured hippocampal neurons from WT and *Lgdel* ^+/−^ mice, albeit these characterizations are beyond the scope of the current work. We quantified the read counts for all genome-aligned miRNA sequences during embryonic developmental phases (8, 16, and 26 DIVs). We found an incremental delay in the expression level of miRNAs in *Lgdel* ^+/−^ neurons over developmental ages, compared to WT neurons, albeit the increase of read counts from 8 to 26 DIVs in both *Lgdel* ^+/−^ and WT neurons (Supplementary Figure S2e). Further, the analysis of small RNA deep sequencing data from 8 to 26 DIVs in *Lgdel* ^+/−^ and WT samples showed a progressive delay in the increase of miRNAs global expression in *Lgdel* ^+/−^ compared to WT. Interestingly, among the most downregulated miRNAs in *LgDel* ^+/−^ samples, in our experimental attempt, we found miR-340–5p, a candidate regulator of NKCC1 expression, as it was highlighted by different prediction software to possibly target *Nkcc1* mRNA. Additionally, we also found miR-101a-3p downregulated in *Lgdel* ^+/−^ compared to WT samples. This is in accordance with a recent study that reported the role of miR-101 to orchestrate early postnatal network development and also to target NKCC1 to facilitate the GABA-signaling switch^34^.

Taken together, these results reveal the higher NKCC1 and the lower KCC2 expressions in embryonic *Lgdel* ^+/−^ neurons compared to WT neurons. Thus, the deregulation of NKCC1 and KCC2 suggests a delayed GABA-switch in *Lgdel* ^+/−^ neurons that may lead to a developmental delay and altered network formation. Given that NKCC1 and KCC2 expressions were also found significantly altered in *Dgcr8* ^+/−^ compared to WT cultures, this may indicate a contribution of the *dgcr8* gene, i.e., inferred by the altered miRNA expression level, to modulate the GABA-switch delay in the early embryonic development of *Lgdel* ^+/−^ neuronal networks.

### Delayed GABAergic-signaling in *Lgdel*^+/−^ and *Dgcr8*^+/−^ embryonic hippocampal neurons correlates with an altered spontaneous network activity

**Figure 3:**
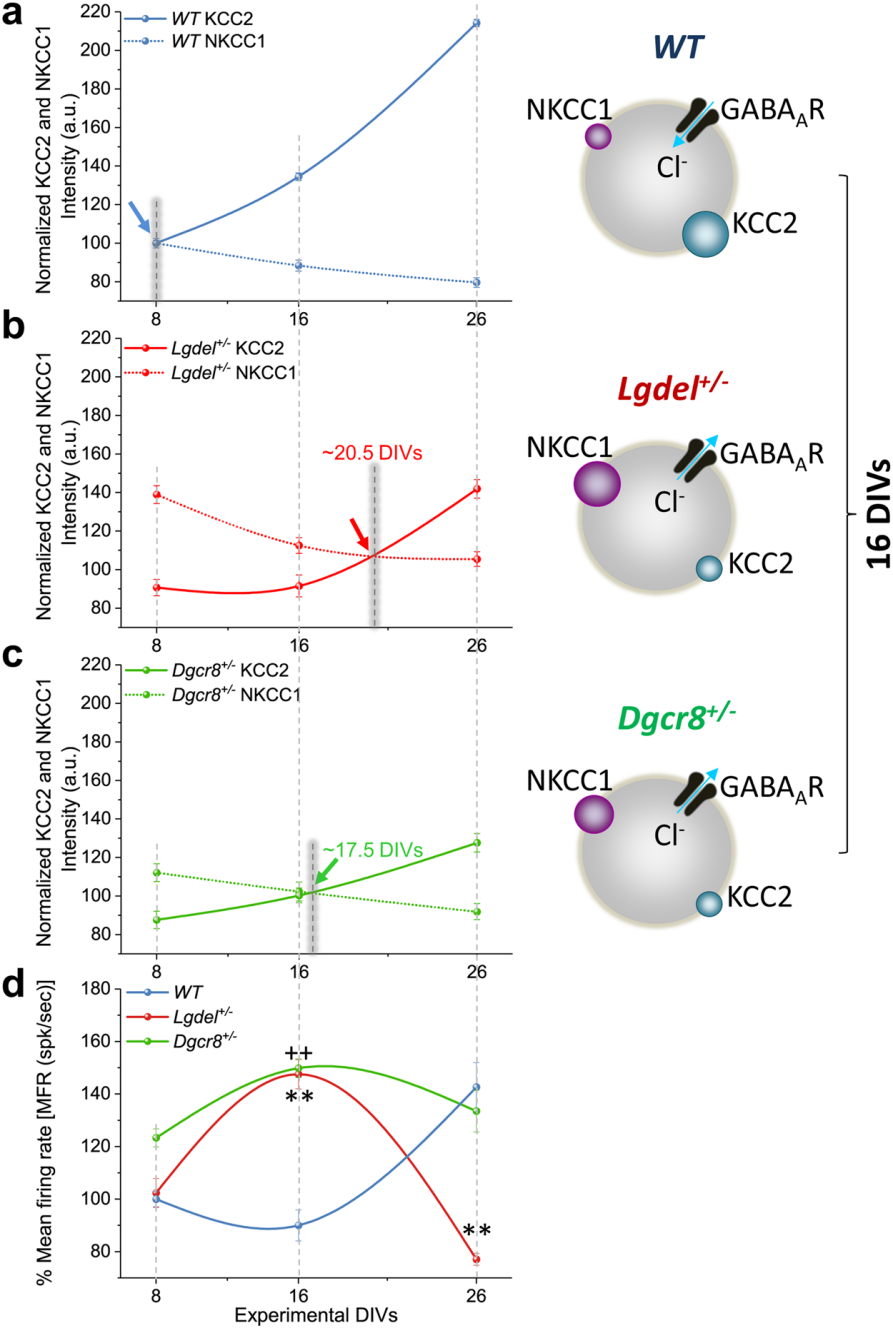
Altered GABAergic-signaling in *Lgdel* ^+/−^ and *Dgcr8* ^+/−^ neurons is correlated with excitable neuronal network firing activity. (**a**) In WT neurons, the quantification of the fluorescence intensities of NKCC1 and KCC2 allows estimating an intersection point that indicates the GABA-polarity switch time point at 8 DIVs. Cartoon (*right up*) illustrates the physiological arrangement (size) of the chloride cotransporters derived from data at 16 DIVs, indicating low and high expression levels of NKCC1 and KCC2, respectively. (**b**) As in (**a**), but for *Lgdel* ^+/−^ neurons, the intersection point corresponding to the GABA-polarity switch is delayed with respect to WT, and it is estimated at ~20.5 DIVs. Cartoon (*right middle*) illustrates the high expression level of NKCC1 and the low expression level of KCC2 observed at 16 DIVs. (**c**) As in (**a**) and (**b**), but for *Dgcr8* ^+/−^ neurons, the GABA-polarity switch is delayed with respect to WT, and it is estimated at ~17.5 DIVs. Cartoon (*right down*) illustrates the arrangement of NKCC1 and KCC2 cotransporters at 16 DIVs. (**d**) The MFR computed from the spontaneous activity of WT, *Lgdel* ^+/−^ and *Dgcr8* ^+/−^ embryonic hippocampal neuronal networks at 8, 16 and 26 DIVs. At 16 DIVs, compared to WT, the hyper-excitable *Lgdel* ^+/−^ and *Dgcr8* ^+/−^ networks showing evident correlation with the expression level of the chloride cotransporters and with the delayed GABA-polarity switches. The hyper-excitable state is indicated by a significantly higher MFR in *Lgdel* ^+/−^ and *Dgcr8* ^+/−^ networks compared to WT networks (**denotes *p* < 0.01 *Lgdel* ^+/−^ vs. WT,++ denotes *p* < 0.01 *Dgcr8* ^+/−^ vs. WT, ANOVA).

Spontaneous electrical neuronal activity is associated with the early development of neural circuits^20,25,35^. Therefore, we examined whether the expression levels of the chloride cotransporters in WT, *Lgdel* ^+/−^ and *Dgcr8* ^+/−^ cultures could be correlated with the spontaneous network activity. In particular, we aimed to pinpoint the developmental GABA-polarity switch during network maturation. We first characterized the biochemical profile of the chloride cotransporters NKCC1 and KCC2 (Fig. 3a–c) and the electrophysiological network activity profile (Fig. 3d) at 8, 16 and 26 DIVs. First, based on the quantitative expressions of immature chloride cotransporters that were described earlier in this study, we generated a single-plot showing the expression of NKCC1 and KCC2 in the different genotypes. In turn, this allowed us to estimate the intersection point that confers the GABA-polarity switch time-point. Next, we correlated the occurrence of the switch with the similar spontaneous network firing activity, as obtained from high-resolution extracellular electrical recordings.

To perform network activity recordings from WT, *Lgdel* ^+/−^ and *Dgcr8* ^+/−^ neuronal cultures at 8, 16, and 26 DIVs, we used CMOS-MEAs^36–38^ with cell-culture and substrate functionalization conditions optimized for mouse primary neuronal cultures (see Methods). These devices provide 4096 closely spaced electrodes for simultaneously recording the spontaneous electrical activity of several thousands of single hippocampal neurons within the network. To characterize the network-wide activity levels we computed the mean firing rate (MFR), and we used this parameter to correlate the activity with the estimated timing of the GABA-polarity switch in the different cultures. In WT cultures, we found a dynamic switch in the action of GABA from excitatory-to-inhibitory that occurred at 8 DIVs, as shown by the intersection of NKCC1 and KCC2 expressions in (Fig. 3a). This GABA-polarity switch time-point was correlated with the increase of the MFR that followed the temporal maturation of the neural network from 8 to 26 DIVs until the complete maturation of the glutamatergic synapses was reached^39^ (Fig. 3d; blue line). On the other hand, it is intriguing to remark that the intersection of NKCC1 and KCC2 in *Lgdel* ^+/−^ cultures was delayed and occurred after 16 DIV (estimated at ~20.5 DIVs) (Fig. 3b). This delayed switch was correlated with the observed excitable network activity indicated by the pinnacle of MFR at 16 DIVs (Fig. 3d; red line). Afterward, the activity in *Lgdel* ^+/−^ cultures progressively declined over 26 DIVs. Finally, results from the intersection of NKCC1 and KCC2 expression levels of *Dgcr8* ^+/−^ cultures elucidated a GABA-polarity switch that also occurred after 16 DIV (estimated at ~17.5 DIVs) (Fig. 3c), slightly before the switch estimated for *Lgdel* ^+/−^ cultures. The switch time-point of *Dgcr8* ^+/−^ cultures was correlated with the pinnacle of MFR at 16 DIVs (Fig. 3d; green line), which is indicative of an excitable network behavior as observed in *Lgdel* ^+/−^ cultures, but minimally subsided to reach a similar level of activity as in WT cultures at 26 DIVs.

Altogether, these results suggest significant developmental delays in networks of embryonic *Lgdel* ^+/−^ and *Dgcr8* ^+/−^ neurons compared to WT. For both *Lgdel* ^+/−^ and *Dgcr8* ^+/−^ cell cultures, this functional derangement in the network activity is correlated with an altered expression of the chloride cotransporters, as indicated by the delayed decrease of NKCC1 expression and the delayed increased of KCC2 expression (Fig. 3, cartoon at 16 DIVs). Our results also unveil the contribution of the *dgcr8* gene deletion to the altered GABA-polarity switch and the electrophysiological network activity observed in the *Lgdel* ^+/−^ cultures.

### Network homeostatic plasticity is impaired in *Lgdel*^+/−^ and attenuated in *Dgcr8*^+/−^ embryonic hippocampal cultures

Network homeostatic plasticity is a key mechanism that allows maintaining a stable, functional state in neural circuits when the network is subdued by developmental or input-driven changes^40^. Homeostatic responses are an essential property of healthy networks. In contrast, the disability of a network to restore similar initial activity levels upon mild extrinsic perturbations (induced by pharmacological manipulations), or intrinsic modifications (neurodevelopmental changes), is an indicator of pathological network impairments. Notably, previous studies reported an age-dependent alteration of synaptic plasticity in adult *Df*(*16*)*A* ^+/*−*^ *and Dgcr8* ^+/−^mice^28,41,42^. We thus asked whether *Lgdel* ^+/−^ and *Dgcr8* ^+/−^ neuronal networks exhibited plasticity deficits also at early neurodevelopmental phases, which in turn, might affect the neuronal maturation.

**Figure 4:**
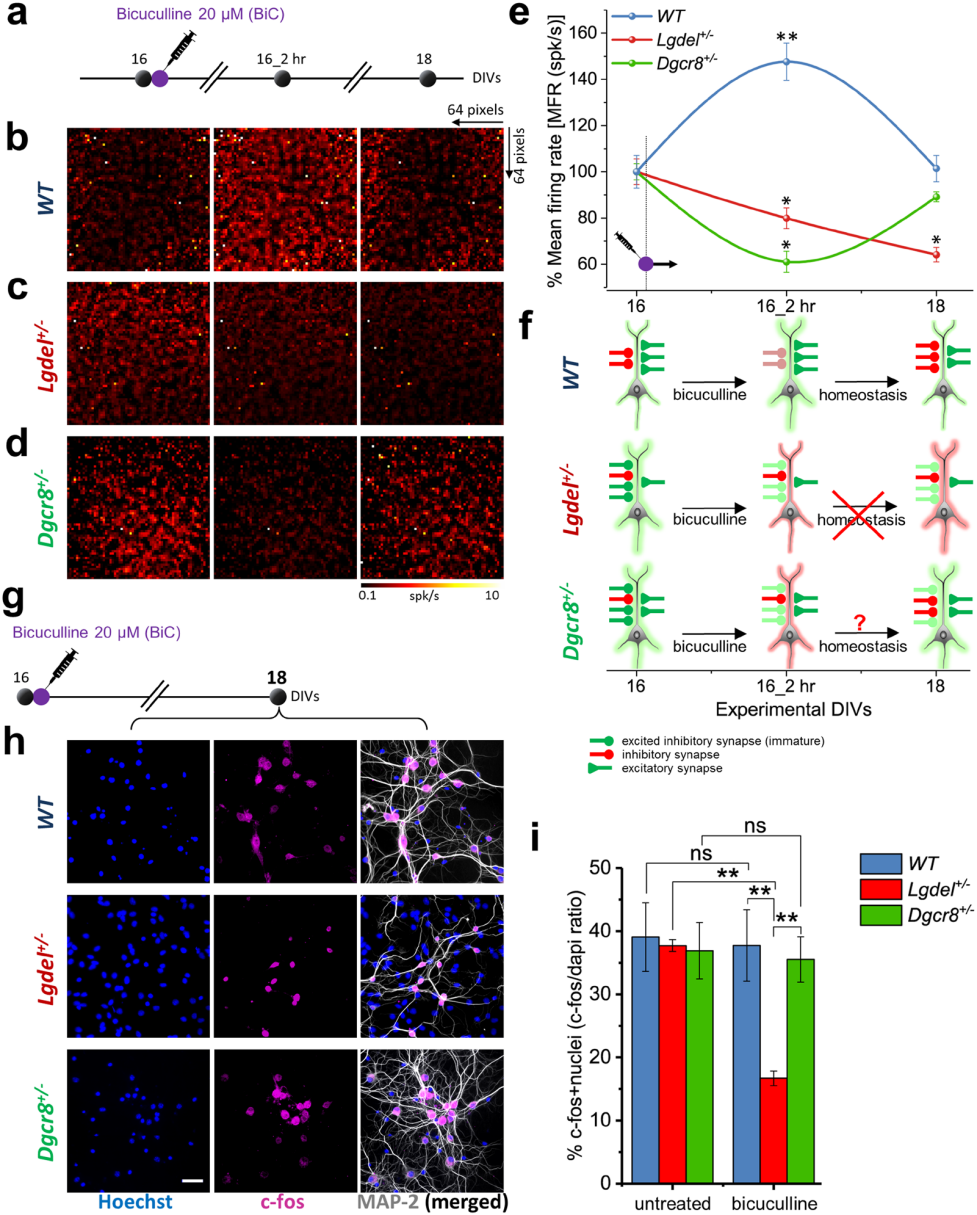
Electrical and optical evidence of dysregulated network homeostatic plasticity in *Lgdel* ^+/−^ and *Dgcr8* ^+/−^ networks. (**a**) Schematic illustration of the experimental protocol used to test network homeostatic plasticity responses upon 20 µM bicuculline-treatment. (**b**) Snapshots showing the homogenous averaged MFR maps (64 × 64 electrode pixels) for a WT network at the different experimental time points and each map is computed for a recording of 10 minutes. (**c**) As in (**b**), but for a *Lgdel* ^+/−^ network. At 16 DIVs the network manifests an already higher averaged MFR than WT. Upon bicuculline-treatment the maps at 16_2 hr and 18 DIVs indicate a decrease of the network firing activity without the homeostatic restoration to the initial condition. (**d**) As in (b) and (c), but for a *Dgcr8* ^+/−^ network. (**e**) Quantification of the MFR shows after bicuculline-treatment (16_2 hr), a significantly decrease in *Lgdel* ^+/−^ and *Dgcr8* ^+/−^ networks, and a significantly increase in WT from the initial baseline (**p < 0.01 WT, *p < 0.05 *Lgdel* ^+/−^, *p < 0.05 *Dgcr8* ^+/−^, ANOVA; n = 5). At 18 DIVs, the MFR continuously decreases in *Lgdel* ^+/−^ networks, while it returns to the baseline value in *Dgcr8* ^+/−^ networks and sets to a nearly similar MFR of WT. (**f**) Cartoon illustrates the homeostatic regulation of the excitation-inhibition balance in WT, *Lgdel* ^+/−^ and *Dgcr8* ^+/−^ neurons at the different time points. (**g**) Schematic of the experimental protocol used to assay the cellular neuronal activity with c-fos immunofluorescence after 48 hr from bicuculline-treatment. (**h**) Fluorescence micrographs showing the c-fos expression 48 hr after bicuculline-treatment in WT, *Lgdel* ^+/−^ and *Dgcr8* ^+/−^ networks and confirming previous electrical read-outs. (**i**) Quantification of c-fos-positive nuclei ratio 48 hr after bicuculline-treatment. Data showing a significant decrease in expression in *Lgdel* ^+/−^ neurons compared to WT, and *Dgcr8* ^+/−^ neurons (**p < 0.01, ANOVA; n = 5), as well as compared to the expression in *Lgdel* ^+/−^ neurons before bicuculline-treatment (untreated) (**p < 0.01, ANOVA, n = 5).

To characterize homeostatic plasticity responses, hippocampal neuronal cultures from WT, *Lgdel* ^+/−^ and *Dgcr8* ^+/−^ mouse embryos were treated at 16 DIVs with 20 μM bicuculline and the network-wide firing activity was then monitored for 48 hr after bicuculline-treatment with CMOS-MEAs recordings (Fig. 4a). As it was previously demonstrated^40^; here, we aimed at using this mild pharmacological manipulation to temporarily destabilize the network by slightly elevating the network firing rate through a small reduction of the GABAergic network inhibition. As expected, WT cultures showed a significant increase in the mean firing activity of the network after 2 hr bicuculline-treatment (147.58 ± 10.05% spk/s; *p* < 0.01, ANOVA) and a return nearly to its baseline after 48 hr (101.38 ± 0.7% spk/s) (Fig. 4b,e blue). In contrast, in *Lgdel* ^+/−^ cultures (Fig. 4c,e red), the network firing activity progressively declined after 2 hr bicuculline-treatment (79.88 ± 4.48% spk/s; *p* < 0.05, ANOVA) and this diminution continued over 48 hr after bicuculline-treatment (64.11 ± 3.13% spk/s; *p* < 0.05, ANOVA) with respect to its baseline firing activity. Remarkably, *Dgcr8* ^+/−^ cultures showed a promiscuous combination of the network firing behavior observed in WT and *Lgdel* ^+/−^ cultures (Fig. 4d,e green). Indeed, *Dgcr8* ^+/−^ cultures responded with a significant decay in activity after 2 hr bicuculline-treatment (61.01 ± 1.56% spk/s; *p* < 0.05, ANOVA), as observed in *LgDel* ^+/−^ networks, and a restored firing activity returning to the baseline after 48 hr bicuculline-treatment (89.12 ± 2.15% spk/s; *p* < 0.05, ANOVA), as yielded in the WT networks. These results reveal the homeostatic dysregulation of *Lgdel* ^+/−^ and *Dgcr8* ^+/−^ networks with respect to WT.

Our results are delineated in (Fig. 4f) showing in WT, the homeostatically regulated firing activity by the cooperation of excitatory (green) and inhibitory (red) synapses. At 16 DIVs *Lgdel* ^+/−^ already holds higher immature excited inhibitory synapses than excitatory synapses, which leads to a reduction of the network firing activity after bicuculline-treatment due to dysregulated homeostatic plasticity. Although *Dgcr8* ^+/−^ at 16 DIVs possess more immature excited inhibitory synapses than excitatory synapses as *Lgdel* ^+/−^, *Dgcr8* ^+/−^ succeeds to return to the baseline network firing activity. This happens because the GABA-polarity switch in *Dgcr8* ^+/−^ neurons occurs earlier than in *Lgdel* ^+/−^ neurons.

Further, to validate at the cellular level these altered homeostatic plasticity responses observed with electrical recordings, we measured by HCI the level of c-fos expression in neurons from WT, *Lgdel* ^+/−^ and *Dgcr8* ^+/−^ cultures at 16 DIVs (baseline) and 18 DIVs (after 48 hr bicuculline-treatment) (Fig. 4g–i and Supplementary Figure S3a,b). Quantifications of these optical readouts revealed the changes in the cellular neuronal activity (Fig. 4i). At 16 DIVs, before bicuculline-treatment, we found similar c-fos-positive nuclei ratios under untreated conditions in WT, *Lgdel* ^+/−^ and *Dgcr8* ^+/−^ cultures (39.06 ± 5.44%, 37.72 ± 0.92% and 36.9 ± 4.48%), respectively (Fig. 4h and Supplementary Figure S3a,b). After 48 hr bicuculline-treatment, the c-fos-positive nuclei ratios in WT and *Dgcr8* ^+/−^ cultures showed a restoration to the untreated baseline condition (37.73 ± 5.65% and 35.51 ± 3.58%), respectively (Fig. 4h,i). In contrast, *Lgdel* ^+/−^ cultures exhibited a significant reduction of c-fos-positive nuclei ratio (16.68 ± 1.16%; *p* < 0.01, ANOVA) compared to their baseline untreated condition (Fig. 4h,i and Supplementary Figure S3a,b). Thus, this significant cellular loss of c-fos-positive neurons in *Lgdel* ^+/−^ cultures confirms the decayed network activity after 48 hr bicuculline-treatment observed with our electrical recordings.

Next, we tested whether homeostatic plasticity deficits in *Lgdel* ^+/−^ and *Dgcr8* ^+/−^ cultures yielded a functional derangement of the network dynamics based on the particular firing frequency of neuronal populations in the network. To do so, we exploited our large-scale electrical recordings to analyze the multiunit distributions of neuronal firing rates using lognormal-like quantification, as previously reported in *in vivo* ^43^ and *in vitro* ^44^ studies. Although there is no discernable boundary between the two sides of a lognormal distribution, a biological notion can be resolved based on the firing frequencies associated with a given neuronal population. Indeed, under physiological conditions, the right-tail of the firing distribution denotes a minority of highly active neurons (interneurons). Given the strong connections of this fast-firing minority population in a network confer stability and transformation of neural information. On the other hand, the left tail of the distribution indicates the firing activity of slow-discharge neurons that may provide excitation and set plastic features to a neural network^45^. In turn, this can provide a balance mechanism to maintain the mean activity level of a self-organized network and to ensure inhibition by counteracting the effects of the fast-firing minority population. We found that after 2 hr bicuculline-treatment, the lognormal-like distribution of WT networks was skewed toward high frequencies compared to the distribution at 16 DIVs, as indicated by the mean parameter of the Gaussian fit (Supplementary Figure S4a,b,d blue). After 48 hr bicuculline-treatment, the distribution of WT networks shifted toward left (low firing frequencies), thus indicating that networks were driven by homeostatic plasticity to stabilize the firing activity to a baseline state similar to the one observed at 16 DIVs (Supplementary Figure S4c,d blue). The peaks of the Gaussian distributions in *Lgdel* ^+/−^ and *Dgcr8* ^+/−^ networks at 16 DIVs already yielded higher frequencies with respect to WT networks (Supplementary Figure S4a,d red, and green). On the contrary to the WT, we found that in *Lgdel* ^+/−^ and *Dgcr8* ^+/−^ networks after 2 hr bicuculline-treatment, the lognormal-like distributions shifted to the left, toward lower frequencies (Supplementary Figure S4b,d red, and green). After 48 hr bicuculline-treatment, the distribution of *Lgdel* ^+/−^ networks shifted further left toward low frequencies, while *Dgcr8* ^+/−^ networks returned nearly to the initial state observed at 16 DIVs (Supplementary Figure S4c,d red, and green). Therefore, right tails of the distributions of *Lgdel* ^+/−^ and *Dgcr8* ^+/−^ networks confirm that GABA is excitatory and in turn, by blocking this neuronal population with bicuculline, the firing activity is decreased, and the distributions shift toward the left.

In sum, these results demonstrate the abnormal firing dynamics of *Lgdel* ^+/−^ and *Dgcr8* ^+/−^ networks associated with dysregulated homeostatic plasticity to set the appropriate synaptic rule to counter the contribution of excitable GABAergic neurons. Further, these responses can be interpreted as a consequence of the altered GABA-polarity switch that we have found in the neuronal populations of *Lgdel* ^+/−^ and *Dgcr8* ^+/−^ networks.

### Synchronous activity is altered in *Lgdel*^+/−^ embryonic hippocampal networks

**Figure 5:**
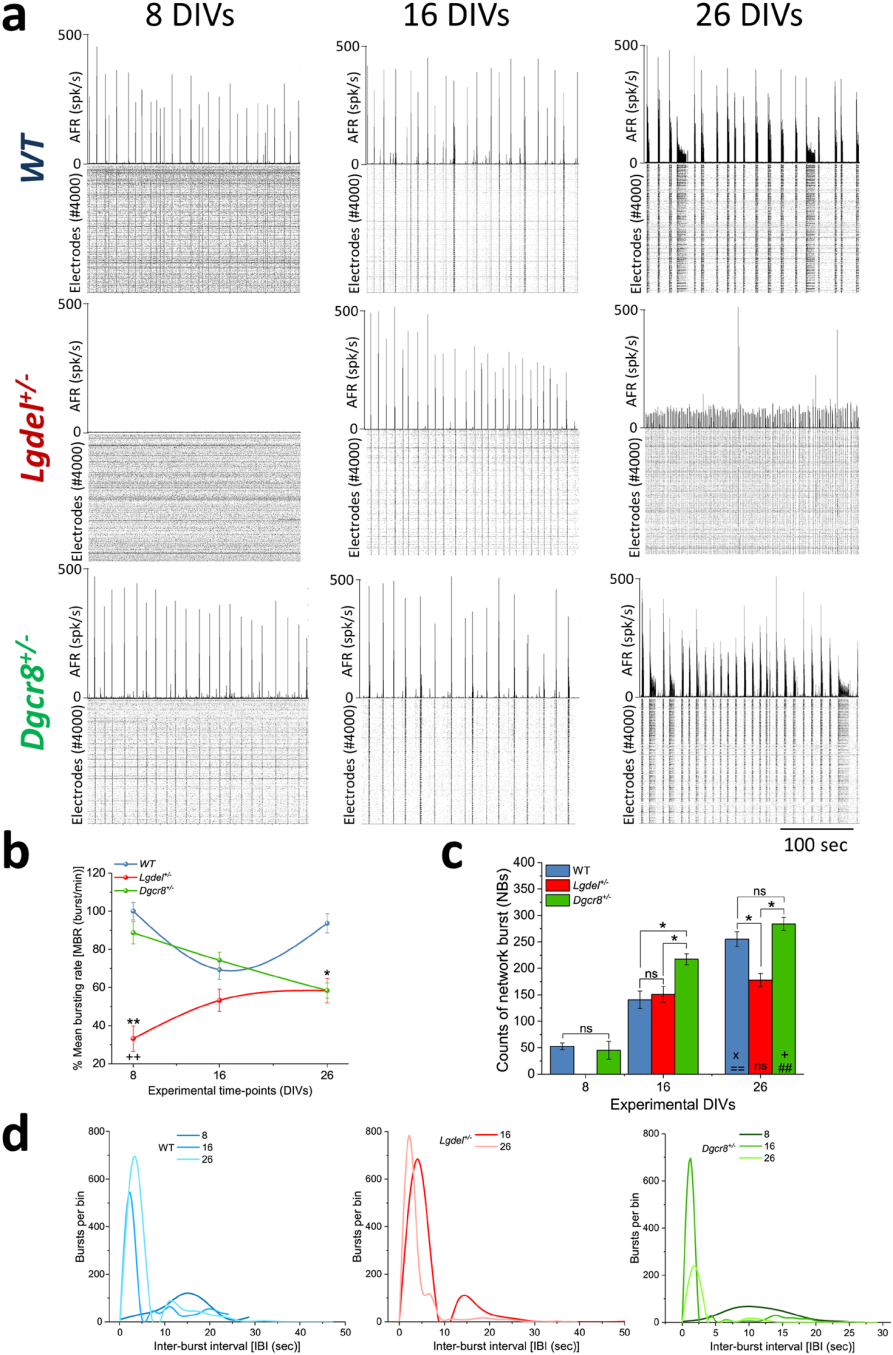
Desynchronization and deregulation of spontaneous bursting activity in *Lgdel* ^+/−^ networks over developmental phases. (**a**) Graphs display the network-wide averaged firing rate (AFR) and raster plots of the spiking activity from recordings in WT, *Lgdel* ^+/−^ and *Dgcr8* ^+/−^ networks (n = 5) at 8, 16 and 26 DIVs. (**b**) MBR indicates the mean bursting rate computed for single electrodes. At 8 DIVs, the MBR displays a significantly lower value in *Lgdel* ^+/−^ network compared to WT and *Dgcr8* ^+/−^ networks (**denotes *p* < 0.01 to WT, ++ denotes *p* < 0.01 to *Dgcr8* ^+/−^, ANOVA). During development, *Lgdel* ^+/−^ networks manifest a tendency to increase the MBR and reach the highest value at 26 DIVs, with a similar MBR to *Dgcr8* ^+/−^ networks, but significantly lower than WT networks (**denotes *p* < 0.01, ANOVA). (**c**) The quantification of NBs shows the lack of *Lgdel* ^+/−^ networks to express these events at 8 DIVs compared to WT and *Dgcr8* ^+/−^ networks. In all networks NBs increase over developmental phases. *Lgdel* ^+/−^ networks reach nearly similar NB counts of WT at 16 DIVs but manifest a significantly lower NB counts than *Dgcr8* ^+/−^ network (*p < 0.01, ANOVA). At 26 DIVs *Lgdel* ^+/−^ networks manifest significantly lower NB counts than WT and *Dgcr8* ^+/−^ networks (*p < 0.01, ANOVA). (**d**) Distribution of IBIs yielded from the temporal classification of bursts in the selected time bins from WT networks. (**e**) As in (d), but for *Lgdel* ^+/−^ networks. (**f**) As in (**d**) and (**e**), but for *Dgcr8* ^+/−^ networks.

Synchronous spiking activity and changes in its dynamics are fundamental for brain circuits development^46^ and count for the critical establishment of synaptic connections^47^ and activity-dependent network formation^48^. Synchronous activity arises from dynamically connected neural assemblies has been postulated to render processing of neuronal information during early developmental phases^49^. A wealth of data suggests that *in vitro* spontaneous events of synchronous spikes (or bursts) expressed by networks formed by embryonic neurons may retain some properties of the early synchronous activity observed *in vivo* ^50^. Here, we investigated whether burst properties varied during the formation of *Lgdel* ^+/−^ and *Dgcr8* ^+/−^ neuronal networks, compared to WT networks (Fig. 5a). In order to characterize the onset of bursts and their properties during development from CMOS-MEAs recordings obtained from these neuronal cultures, we quantified the mean bursting rate (MBR) (Fig. 5b), the average count of network-wide burst events (NBs) (Fig. 5c) and the distributions of inter-burst intervals (IBIs) (Fig. 5d). The MBR was computed from all single active electrodes that were recording bursts during sessions of 10 minutes; the NBs confer the level of temporal synchronization over a 100 ms time-window, and the IBIs characterize the mean time-distance between each subsequent NB event.

In WT and *Dgcr8* ^+/−^ networks, we observed the first network-wide synchronous spiking events (NBs) at 8 DIVs, whilst *Lgdel* ^+/−^ networks did not show any NB event at this age (Fig. 5a and Supplementary movies), albeit minimal single-electrode bursting activity as indicated by the MBR value (Fig. 5b). During later stages of development, the spiking rate increased in all networks, and the dynamics of their activity changed, showing a different degree of burstiness, in addition to the temporal clustering of the burst events^51^. At 16 DIVs, regular network-wide bursts were observed in WT, *Lgdel* ^+/−^ and *Dgcr8* ^+/−^ cultures (Fig. 5a). Notably, at 26 DIVs WT and *Dgcr8* ^+/−^ cultures showed a particular change in their bursting pattern and expressed NBs that occurred over short-time periods separated by prolonged silent time windows (Fig. 5a). This dynamic was not observed in *Lgdel* ^+/−^ cultures that remained expressing consistent bursting patterns, but with a lower spiking rate compared to events at earlier DIVs (Fig. 5a). As shown in (Fig. 5b,c), the quantification of the burst parameters at 8 DIVs in *Dgcr8* ^+/−^ networks showed very similar MBR (88.72 ± 5.86% burst/min) and NBs (45 ± 16.89%) compared to WT networks. In contrast, *Lgdel* ^+/−^ networks showed a significantly lower MBR (33.16 ± 6.7% burst/min; *p* < 0.01, ANOVA) compared to WT and *Dgcr8* ^+/−^ networks and lacked the NBs. At 16 DIVs, *Lgdel* ^+/−^ networks showed nearly similar MBR (53.24 ± 5.87% burst/min) compared to WT and *Dgcr8* ^+/−^ networks, as well as similar NBs to WT, but 1.6-fold lower NBs compared to *Dgcr8* ^+/−^ networks. At 26 DIVs, the MBR increased significantly for WT networks compared to *Lgdel* ^+/−^ and *Dgcr8* ^+/−^ networks (93.7 ± 5.03% burst/min; *p* < 0.05, ANOVA), while the NBs for WT and *Dgcr8* ^+/−^ networks significantly increased compared to *Lgdel* ^+/−^ networks. Noticeably, the IBIs changed over development. At early network developmental phases, the IBIs were marked with widely temporally distributed bursts, while at older stages they were characterized by bursting dynamics that changed into more narrowly distributed events (Fig. 5d). At 26 DIVs, the network-wide synchronized events recorded from WT and *Dgcr8* ^+/−^ cultures appeared as “superburst-like patterns” (Fig. 5a). Interestingly, these bursting patterns were previously associated with a higher level of network maturation in rodent neuronal cultures^51^. In contrast, *Lgdel* ^+/−^ networks did not show any superburst-like events and remained expressing fixed-size bursts; thus, indicating a lower level of maturation on both WT and *Dgcr8* ^+/−^ cultures (Fig. 5a).

Taken together, these results indicate that networks formed by *Lgdel* ^+/−^ embryonic neurons show a significant delay in the expression of synchronous spiking activity and the developmental changes of the dynamics of these events with respect to WT and *Dgcr8* ^+/−^ networks. Importantly, this might indicate the presence of severe alterations in the activity-dependent formation of neuronal networks in *Lgdel* ^+/−^ models during their early development.

### Morphological alterations in *Lgdel*^+/−^ and *Dgcr8*^+/−^ embryonic hippocampal neurons

**Figure 6:**
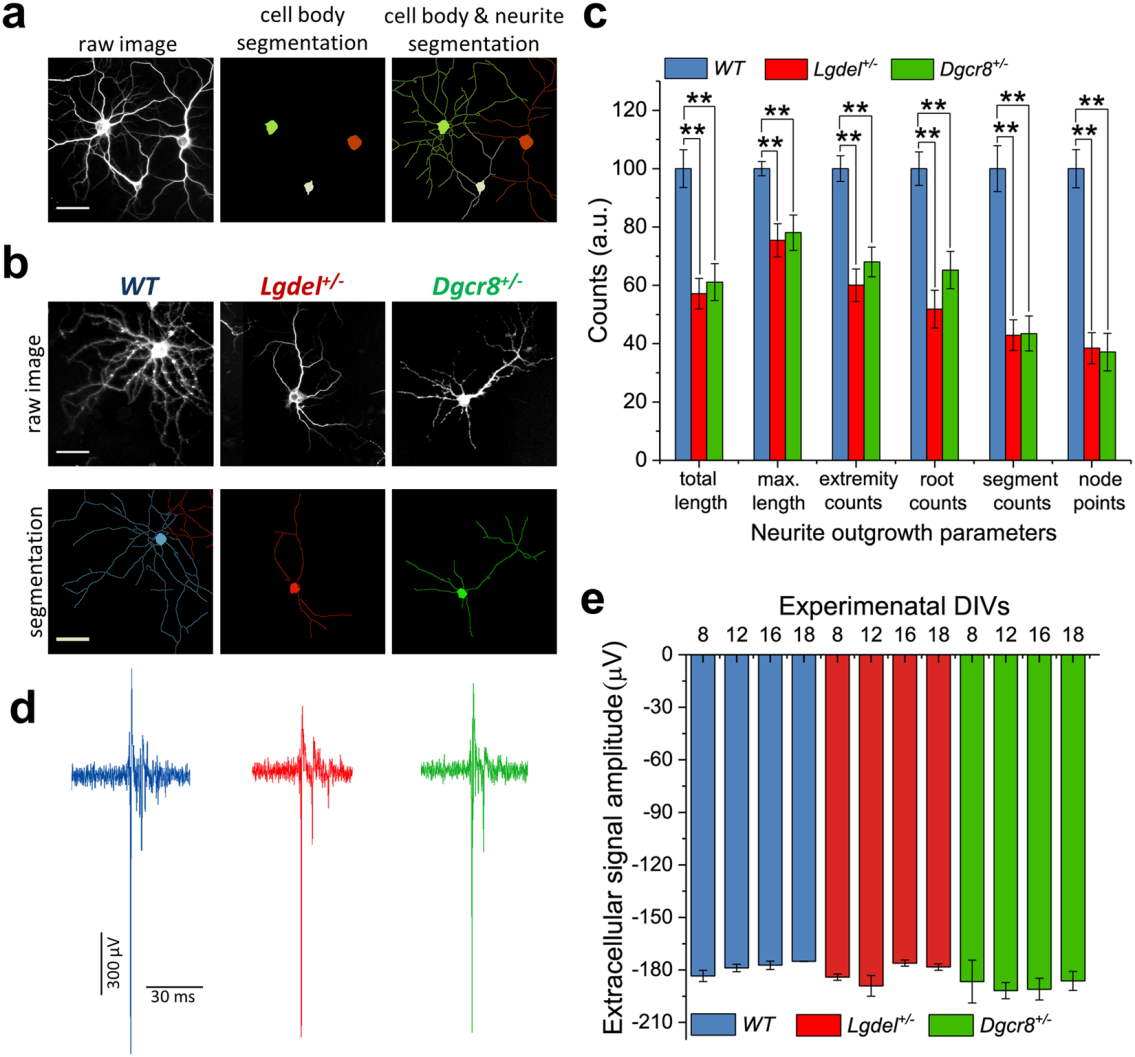
Neurite outgrowth measurements unveil alteration in neuromorphogenesis in *Lgdel* ^+/−^ and *Dgcr8* ^+/−^ neurons. (**a**) Micrographs showing the raw image of WT neurons (MAP-2) and the segmentation mask for cell bodies (Hoechst) that are used by the neurite outgrowth algorithm to obtain the final cell body and neurite segmentation masks. Scale bar represents 30 µm. (**b**) Micrographs of WT, *Lgdel* ^+/−^ and *Dgcr8* ^+/−^ neurons before segmentation (raw images) and after final segmentation. Scale bar is 30 µm. (**c**) Quantifications of neurite outgrowth measurements from WT, *Lgdel* ^+/−^ and *Dgcr8* ^+/−^ neurons (n = 5 for). Statistical comparisons confirm that all parameters’ values in *Lgdel* ^+/−^ and *Dgcr8* ^+/−^ neurons are significantly lower than WT neurons (**p < 0.01, ANOVA). (**d**) Signal traces of typical spontaneous spikes extracellularly recorded from WT, *Lgdel* ^+/−^ and *Dgcr8* ^+/−^ cultures at 8 DIVs. (**e**) Quantifications of the negative peaks of the spontaneous extracellular spikes recorded from WT, *Lgdel* ^+/−^ and *Dgcr8* ^+/−^ cultures (n = 5) at different time points (8, 12, 16, 18 DIVs). This analysis reveals the non-significant difference of the spike amplitudes between the genotypes.

Evidence in adult animals has been found for abnormalities in spine morphogenesis that ascribed to the deficits of structural synaptic plasticity and neuronal morphological alterations in the 22q11.2 microdeletion mouse model^52,53^. Here, we aimed at assessing whether, at very early embryonic developmental stages, *Lgdel* ^+/−^ and *Dgcr8* ^+/−^ networks exhibited structural deficits in features of neurite outgrowth that could be associated with the observed functional impairments. We performed morphological measurements of neurite processes by using an automated fluorescence HCI system on WT, *Lgdel* ^+/−^ and *Dgcr8* ^+/−^ embryonic hippocampal cultures at 5 DIVs. The acquired images were quantified based on the detection of two fluorescence channels (Fig. 6a). We quantified the neuritogenesis parameters and generated a series of measures on the neurite outgrowth.

Our results showed that all measures of the averaged neurite outgrowth parameters were significantly reduced in both *Lgdel* ^+/−^ and *Dgcr8* ^+/−^ cultures compared to WT cultures (*p* < 0.01, ANOVA) (Fig. 6b,c). Theoretical and experimental approaches have suggested that neuritogenesis plays a role in modulating synaptic efficacy and in the generation of spiking activity^54,55^. Thus, we next examined whether alterations in neurite morphology at early onset (5 DIVs) was accompanied by changes in the amplitude of the spontaneous firing activity in later developing phases (8, 12, 16 and 18 DIVs). To do so, we analyzed the averaged extracellular signals-amplitudes that were recorded from WT, *Lgdel* ^+/−^ and *Dgcr8* ^+/−^ networks. This analysis was performed with a highly conservative thresholding method that was assessed by pre-selecting three different signal amplitude values. Further, the average of signal amplitudes was computed for all extracellular signals that were larger than (−250 μV). However, despite the observed morphological differences, the signal amplitude analysis showed no significant difference between WT, *Lgdel* ^+/−^ *and Dgcr8* ^+/−^ (Fig. 6d,e). On the whole, these results suggest that the structural deficits observed in *Lgdel* ^+/−^ *and Dgcr8* ^+/−^ cultures might be a consequence of the network activity dysfunctions, resulting from dysregulated expression of the chloride cotransporters, rather than being their source.

### Bumetanide restores physiological network behavior and rescues homeostatic deficits in *Lgdel*^+/−^ embryonic hippocampal neurons

Recently, a growing body of evidence suggests that the NKCC1 antagonist bumetanide, an already FDA approved drug^56^, attenuates the intracellular chloride concentration [Cl^−^]_int._ during the excitatory action of GABA responses in immature neurons, thus producing a negative shift in reversal potential of the GABA_A_ receptor (E_GABA_). Noticeably, when bumetanide is administrated in a concentration range of 2–10 μM, it selectively inhibits NKCC1 without affecting KCC2^57^. Therefore, we evaluated whether 10 μM of bumetanide could consistently suppress the high activity of NKCC1 observed in *Lgdel* ^+/−^ immature networks. The rescuing capability of bumetanide was tested both on the spontaneous network-wide activity and to the homeostatic network response induced by bicuculline.

**Figure 7:**
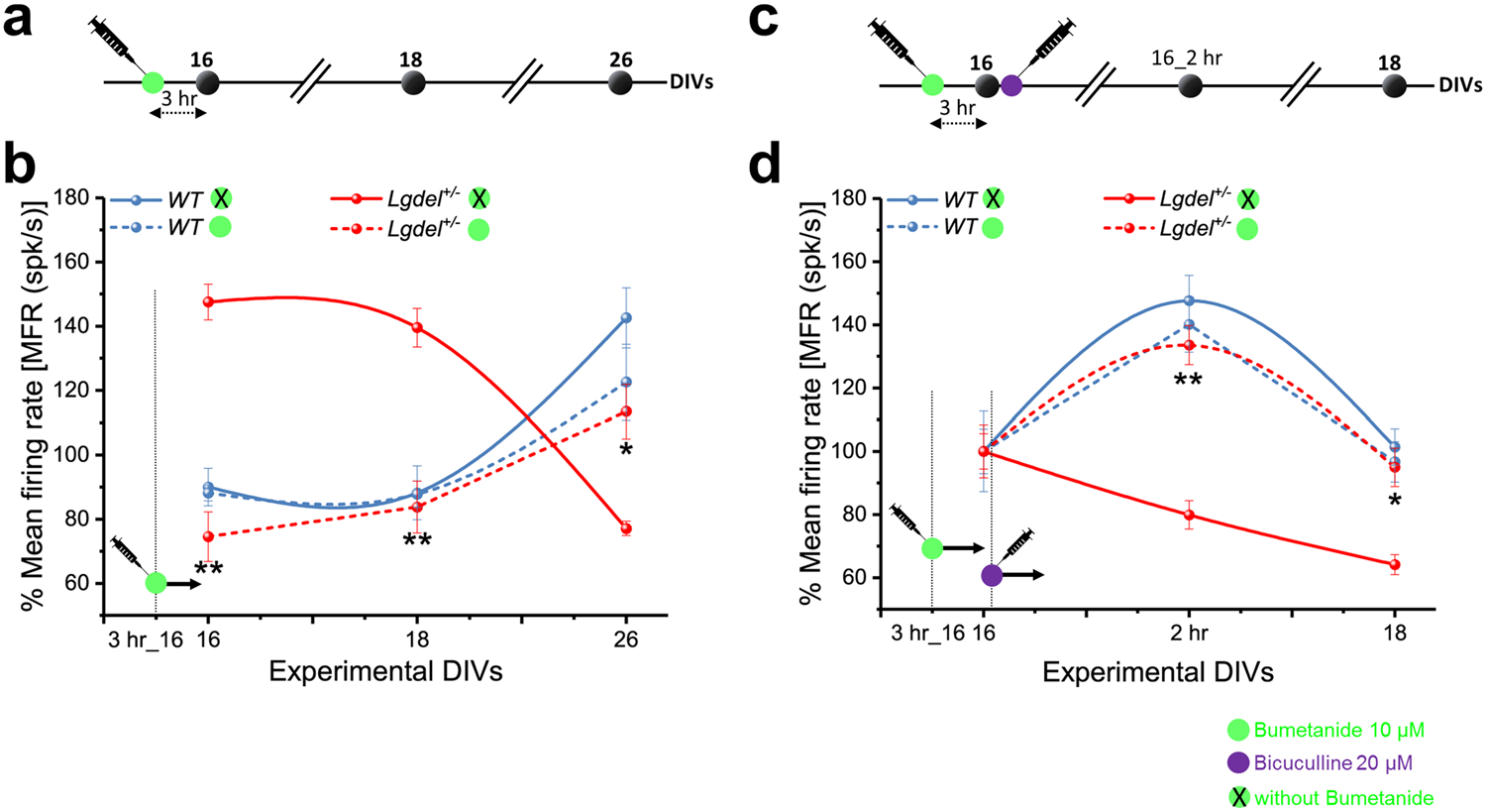
Bumetanide rescues the abnormally excitable *Lgdel* ^+/−^ neuronal network and restores physiological homeostatic plasticity. (**a**) Schematic summary of the experimental protocol used to test the effect of 10 µM bumetanide (administrated 3 hr before the baseline recordings at 16 DIVs) to rescue the spontaneous hyper-excitable activity in *Lgdel* ^+/−^ networks. (**b**) MFR indicates the spontaneous firing activity of WT and *Lgdel* ^+/−^ networks across the developmental phases from 16 to 26 DIVs. After 3 hr from bumetanide-treatment, *Lgdel* ^+/−^ cultures at 16 DIVs exert 2-fold decrease of MFR compared to untreated *Lgdel* ^+/−^ cultures (**p < 0.01, ANOVA; n = 5) and express a nearly analogous MFR as WT cultures. At 18 DIVs, the MFR in bumetanide-treated *Lgdel* ^+/−^ cultures remains significantly low (1.67-fold) compared to the untreated cultures (**p < 0.01, ANOVA; n = 5). At 26 DIVs, bumetanide-untreated *Lgdel* ^+/−^ cultures exert a steep decrease in the MFR, while the BUM-treated *Lgdel* ^+/−^ cultures display 1.47-fold significant recovery (**p < 0.05, ANOVA; n = 5). (**c**) Schematic summary of the experimental protocol used to test the effect of 10 µM bumetanide (administrated 3 hr before the baseline recordings at 16 DIVs) to rescue dysregulated homeostatic plasticity response in *Lgdel* ^+/−^ networks upon 2 hr of 20 µM bicuculline-treatment. (**d**) At 16 DIVs, 5 hr after bumetanide-treatment and 2 hr after bicuculline, *Lgdel* ^+/−^ networks display 1.67-fold significant increase of MFR compared to bumetanide-untreated bicuculline-treated *Lgdel* ^+/−^ cultures (**p < 0.01, ANOVA; n = 5), and showing a nearly analogous MFR as WT cultures. At 18 DIVs, bumetanide-treated *Lgdel* ^+/−^ cultures display a complete restoration to the baseline activity recorded at 16 DIVs (prior bicuculline-treatment) compared to bumetanide-untreated *Lgdel* ^+/−^ cultures (*p < 0.01, ANOVA; n = 5).

In the first set of experiments (Fig. 7a), we applied bumetanide at 16 DIVs to the cell culture media and evaluated its effect on the network-wide spontaneous activity after 3 hr. We found the MFR reduced by 2-fold in bumetanide-treated *Lgdel* ^+/−^ cultures (74.53 ± 7.69%; *p* < 0.01, ANOVA) compared to untreated *Lgdel* ^+/−^ cultures (147.51 ± 5.58%). In WT cultures treated with bumetanide, the spiking activity (88.17 ± 2.46%) remained similar to one of the bumetanide-untreated (89.98 ± 12.86%) samples. At 18 DIVs, the same *Lgdel* ^+/−^ cultures that were previously treated with bumetanide showed 1.67-fold lower mean firing rate (83.76 ± 8.05%; *p* < 0.01, ANOVA) than untreated *Lgdel* ^+/−^ samples (139.53 ± 1.03%). In WT cultures we observed nearly identical mean spiking activity levels both for bumetanide-treated (87.80 ± 4.14%) and untreated (88.21 ± 14.38%) samples, respectively. At 26 DIVs, bumetanide-untreated *Lgdel* ^+/−^ cultures showed a dramatic decrease in activity (83.76 ± 8.05%) compared to WT networks, while bumetanide-treated *Lgdel* ^+/−^ cultures displayed 1.47-fold significant recovery (113.4 ± 8.45; *p* < 0.05, ANOVA) compared to *Lgdel* ^+/−^ untreated cultures and a MFR comparable to WT cultures.

In the second set of experiments (Fig. 7b), we evaluated the effects of bumetanide to rescue response of homeostatic *Lgdel* ^+/−^ networks. To do so, at 16 DIVs and immediately after baseline spiking activity recordings, 10 μM bumetanide was added to *Lgdel* ^+/−^ and WT cultures and left in the bath for 3 hr. Then, 20 μM bicuculline was co-administrated for 2 hr, and the network activity was recorded. Similar to WT networks, we found that the firing activity in *Lgdel* ^+/−^ cultures treated with bumetanide and bicuculline increased 1.67-fold (133.55 ± 6.19%; *p* < 0.01, ANOVA) compared to bumetanide-untreated *Lgdel* ^+/−^ cultures (79.88 ± 4.48%). We observed ostensibly similar spiking activity in bumetanide-treated (140.06 ± 8.74%) and bumetanide-untreated (147.58 ± 0.05%) WT networks under bicuculline, respectively. Remarkably, at 18 DIVs the network activity of bumetanide-treated *Lgdel* ^+/−^ was completely returned to the baseline activity recorded at 16 DIVs (94.95 ± 11.09%; *p* < 0.01, ANOVA), similarly as bumetanide-treated (96.7 ± 6.35%) and bumetanide-untreated (101.38 ± 1.7%) WT samples. On the other hand, bumetanide-untreated *Lgdel* ^+/−^ cultures showed a persistent decrease in the spiking activity that reached a 1.5-fold lower level than bumetanide-treated *Lgdel* ^+/−^ cultures.

In summary, by regulating the activity of the chloride cotransporter NKCC1, our results on bumetanide-treated *Lgdel* ^+/−^ cultures suggest that the altered expression level of NKCC1 is the molecular mechanism responsible for the delayed network activity development and impaired network homeostatic plasticity observed in *Lgdel* ^+/−^networks. This delineates the potential therapeutic effect of bumetanide to restore normal embryonic development in 22q11.2 DS.

## Discussion

Several studies on adult 22q11.2 DS patients have suggested neurodevelopmental abnormalities playing an important role in the pathogenesis of this syndrome. Additionally, the last decade has witnessed a surge of interest in investigating the role of GABAergic-signaling in several neurodevelopmental disorders^8–10,58^, including schizophrenia, which is a neuropsychiatric disorder associated with a high genetic risk for 22q11.2 DS. Importantly, defects of GABAergic-neurotransmission observed in adult patients with the 22q11.2 DS may also be related to aggravation of GABAergic-signaling during early development, but this hypothesis was not yet examined at embryonic developmental stages. Interestingly, GABAergic-interneurons distribution was reported to be significantly altered in adult *Lgdel* ^+/−^ mouse brain circuits^59^.

In this work, we examined GABAergic-signaling in *in vitro* networks prepared from embryonic hippocampal neurons from *Lgdel* ^+/−^ and *Dgcr8* ^+/−^ mice embryos. We monitored the level of expression of the cation-chloride cotransporters NKCC1 and KCC2, the associated electrophysiological developmental profile, and we characterized homeostatic network responses to mild perturbations induced by small doses of bicuculline.

Our results reveal for the first time a critical developmental delay in embryonic networks of *Lgdel* ^+/−^ and *Dgcr8* ^+/−^neurons. We observed alterations in the expression of NKCC1 and KCC2 cotransporters and the association of these expression levels with a hyper-excitability of the network. These deregulated expressions reveal an altered timing of the GABA-polarity switch and correlate with a delay in the functional maturation of the networks. Our data pinpoint specific time switches occurring at ~20.5 DIVs for *Lgdel* ^+/−^ neuronal networks and at ~17.5 DIVs for *Dgcr8* ^+/−^ neuronal networks, thus occurring significantly later compared to WT networks, switching earlier than 8 DIVs. Alterations in the GABA-polarity switch have previously been documented to impact the balance between GABAergic and Glutamatergic transmission in neural networks^60^. Central to these findings is the indication on the early depolarization of GABA-releasing synapses to drive the development of Glutamatergic synapses^61^, where the latter was shown to be reduced in *Df*(16)*A* ^+/−^ mouse model (i.e., a multigene deletion mouse model that spans a segment syntenic to the 1.5-Mb human 22q11.2 microdeletion and encompasses 27 genes)^52^. Within this line of evidence, our results explicitly elucidate the analogues deregulation in the network-wide spontaneous activity mandated by hyper-excitable *Lgdel* ^+/−^ and *Dgcr8* ^+/−^ networks compared to the WT ones. We also found additional evidence on the delayed developmental profile of *Lgdel* ^+/−^ networks by analyzing the spontaneous synchronous network activity. Indeed, *Lgdel* ^+/−^ networks at 8 DIVs express asynchronous spikes, while WT and *Dgcr8* ^+/−^ networks already exert synchronous activity at this early stage of network development. At older developmental stages (26 DIVs), *Lgdel* ^+/−^ networks also express synchronous activity, but display dynamics corresponding to an early stage of maturation compared to WT and *Dgcr8* ^+/−^ networks. We postulate that these results on *Lgdel* ^+/−^ networks likely arise from the overall delayed NKCC1 and KCC2 expression profile that induces an immature balance of excitation-inhibition^62^.

As shown in our results, the delayed GABA-polarity switch also has implications on establishing homeostatic plasticity properties during early phases of network development. An extensive literature highlights the essential role of homeostatic plasticity to adjust the network firing rate dynamically and to maintain neuronal network stability^40^. While WT networks at 16 DIVs exerted an increased excitation in response to bicuculline, we observed an impaired network homeostatic plasticity in *Lgdel* ^+/−^ cultures. These networks are unable to reset to the baseline activity levels and to counteract the dysfunction of inhibitory circuits that still operate as excitatory due to the chloride cotransporters alterations. On the other hand, the homeostatic response of *Dgcr8* ^+/−^ networks was found altered with respect to WT, but it was not completely disrupted as for *Lgdel* ^+/−^ networks. The alteration is likely due to a slightly earlier GABA-polarity switch in *Dgcr8* ^+/−^ networks than *Lgdel* ^+/−^ networks. Cellular quantifications of the c-fos-positive nuclei and the analysis of the dynamical shift of lognormal-like distributions of the neuronal firing frequencies further confirm our results on the electrically characterized homeostatic response.

Given the compelling evidence from some converging experiments on the 22q11.2 mouse model that suggested deficits in the synaptic connectivity and spine morphogenesis^52,53^, we also assessed the presence of abnormal neuritogenesis in *Lgdel* ^+/−^ and *Dgcr8* ^+/−^ networks. In both conditions, we found these abnormalities by multi-parameter analysis obtained with unbiased automated image-based assays. Interestingly, these structural alterations may arise from the network dysfunction per se rather than being the cause of these malfunctions^63^. To investigate this, we compared the amplitude of the spikes over development (8, 16, 18 DIVs) for WT, *Lgdel* ^+/−^, and *Dgcr8* ^+/−^ networks. Our data show a non-significant change in the spiking amplitude, despite the neuritic structure abnormalities. These results suggest that abnormal neuritogenesis in *Lgdel* ^+/−^ and *Dgcr8* ^+/−^ networks might be a consequence of the network dysfunction.

To attenuate the excitable network-wide spontaneous activity and to attempt rescuing the neuronal chloride homeostatic deficits, we treated the *Lgdel* ^+/−^ immature networks with 10 μM bumetanide^14^. Our results demonstrate that this treatment can rescue both the activity levels and homeostatic responses of *Lgdel* ^+/−^ networks with respect to WT. The treatment effect does not only confirm that our results on the network dysfunctions are essentially due to the alteration of the GABAergic-signaling switch but importantly, it also indicates the first time the possible therapeutic effects of blocking the high activity of NKCC1 in early developing networks of the 22q11.2 DS. Remarkably, the bumetanide concentration used in our study selectively inhibits NKCC1 hyperactivity in *Lgdel* ^+/−^ networks and shows no effects on the network activity and homeostatic response of WT neuronal networks. In turn, this indicates the GABA-dependent hyper-excitability mechanism of bumetanide.

Our study also reveals a significant, yet not exclusive contribution of the *dgcr8* gene haploinsufficiency in the GABA-polarity switch and electrophysiological phenotypes observed in the *Lgdel* ^+/−^ neuronal networks. *Dgcr8* gene (one of the deleted genes in the 22q11.2 microdeletion) is essential for the biogenesis of miRNAs, and it exerts miRNA-independent gene-regulatory functions^29^. Noticeably, miRNAs fulfills various functions in neuronal development^64^, plasticity, and neurological diseases^65^. In this context, compelling evidence identified altered miRNA biogenesis associated with the 22q11.2 microdeletion in *Df*(16)*A* ^+/−^ adult mice (8 week old), which was shown to rise due to *dgcr8* gene deficiency^26^. Although no study has shown such alterations during embryonic development in *Lgdel* ^+/−^ neuronal networks, we attempted to assess the miRNA expression profiles in cultured hippocampal neurons from WT and *Lgdel* ^+/−^ mice. Our results showed an incremental delay in the expression level of miRNAs in *Lgdel* ^+/−^ neurons over developmental ages, compared to WT neurons, albeit the increase of read counts from 8 to 26 DIVs in both *Lgdel* ^+/−^ and WT neurons. In turn, the altered expression of miRNAs may play a role, at least partly, in the functional phenotypes observed in our study. Indeed, such as a post-transcriptional regulation could explain the distinct alteration in NKCC1 and KCC2 protein levels rather than in their mRNA levels. Also, given that *Dgcr8* ^+/−^ cultures display intermediate phenotypes between WT and *Lgdel* ^+/−^ cultures, it is tempting to speculate, at least partly, the causative effects of *dgcr8* deletion, and in turn, the miRNAs downregulation, on the delayed timing of the GABA-switch observed in *Lgdel* ^+/−^ cultures. This notion is further corroborated by our recent study that reported the evidence of influential alternative miRNA-independent functions of *dgcr8* during brain development on developmental-related gene expression^66^. Thus, the *dgcr8* haploinsufficiency may dysregulate the gene expression of key genes necessary for the developmental excitatory-to-inhibitory GABA-polarity switch in embryonic *Lgdel* ^+/−^ networks. To this end, our results on *Dgcr8* ^+/−^ neurons reveal the contribution of the *dgcr8* gene haploinsufficiency to the GABAergic-signaling deficits in *Lgdel* ^+/−^ networks, but also indicate the need for extensive future work to characterize the contribution of other deleted genes in *Lgdel* ^+/−^ model. Further, a next challenge will be to identify a possible link of GABA-signaling dysregulation to neuronal activity-regulated transcriptional genes^67^ in *Lgdel* ^+/−^ networks.

Lumping together, the implications of our findings on the delay of GABA-signaling, electrophysiological developmental profile and homeostatic plasticity defects that occur in the early embryonic developmental phases in *Lgdel* ^+/−^ and *Dgcr8* ^+/−^ networks, are tempting to speculate that these deficits might operate the kernel of functional and structural abnormalities that affect adult animal models of the 22q11.2 DS. Prospectively, our *in vitro* study suggests a possible direction for further *in vivo* studies to assess the potential rescue of bumetanide and the contribution of different deleted genes in the 22q11.2 DS to the GABA-signaling properties of neuronal systems. Additionally, 22q11.2 DS on-CMOS-MEA chips combined with HCI may offer an excellent *in vitro* model and readouts to accelerate the probing of the genetic, biology, and of the electrical physiology of the 22q11.2 DS-associated neurodevelopmental disorders, before *in vivo* studies and clinical investigation.

## Methods

### CMOS-MEAs and acquisition system

We performed all electrical recordings using a custom-built acquisition platform based on components of the BioCam system (3Brain GmbH). We grew mouse neuronal hippocampal cultures on CMOS-MEAs (BioChip 4096S+ from 3Brain GmbH) that integrate an array of 4096 recording electrodes (21 × 21 μm^2^ in size, 42 μm pitch) on an active area of (2.67 × 2.67 mm^2^) centered in a working area of (6 × 6 mm^2^). We used the BrainWave software (3Brain GmbH) for data recording and spike detection.

### Preparation and coating of CMOS-MEAs

We sterilized CMOS-MEA devices thoroughly outside the cell culture chamber ring using tissue moistened with EtOH 96%. We placed each device into a sterile 100 × 20 mm Petri dish (Corning) and sterilized the entire culture chamber by filling it with 70% EtOH. After 20 minutes, we rinsed the CMOS-MEAs 4x with sterile double-distilled water (DDW) and dried them under a sterile laminar flow hood. Chips were pre-conditioned by overnight incubation at 37 °C and 5% CO_2_ with Complete Neurobasal Media (CNM) containing 2% B-27 1% penicillin/streptomycin and 1% GlutaMax supplements (all reagents from Life Technologies). The next day, CNM was aspirated, and chips were immediately coated with 50 μg/ml poly-DL-ornithine (PDLO) (Sigma-Aldrich) and incubated overnight at 37 °C and 5% CO_2_. We filled a 35 × 10 mm Petri dish (Corning) with sterile DDW and placed it inside the Petri dish (besides the chip) both to maintain proper humidity and to avoid evaporation of the coating reagents overnight. The next day, CMOS-MEAs were rinsed 4x with sterile DDW and left to dry under the hood before cell seeding.

### Mouse lines and genotyping

Mice were housed under standard conditions in the animal facility at the Fondazione Istituto Italiano di Tecnologia (IIT). All experiments and procedures were approved by the Italian Ministry of Health and IIT Animal Use Committee, by the Guide for the Care and Use of Laboratory Animals of the European Community Council Directives. *Lgdel* ^+/−^ mice (DGS model^27^) carrying a hemizygous deletion from *Idd* to *Hira* (*Lgdel* ^+/−^), were maintained on a C57BL/6 J (Charles River) background. *Lgdel* ^+/−^ mice were crossed with C57BL/6 J mice. The yielded WT (control) and *Lgdel* ^+/−^ embryos were used for the experiments. The conditional heterozygous ablation of *dgcr8* gene was performed by crossing *dgcr8* ^*flox*/*flox*^ mice with *Emx1* ^*Cre*/*wt*^ knock-in mice^66^. Embryos yielded from these crossings were used for the experiments: *dgcr8* ^*flox*/*wt*^/*Emx1* ^*wt*/*wt*^ (WT, controls), *dgcr8* ^*flox*/*wt*^/*Emx1* ^*Cre*/*wt*^ (*dgcr8* ^+/−^conditional *dgcr8* heterozygous). All primers used for the genotyping procedures are listed in Table 1.

### Preparation of primary hippocampal cultures from mouse embryos

We obtained primary hippocampal neurons from brain tissues of embryos of WT, *Lgdel* ^+/−^, and *Dgcr8* ^+/−^ mice at day 18 (E18) using a similar previously described method on rat cultures^68^. All work with primary cultures was performed in accordance with the Italian guidelines and regulations, and all animal procedures carried out in this work were approved by the institutional (IIT) Ethics Committee and by the Italian Ministry of Health and Animal Care (Authorization No. 057/2013 and 214/2015). Briefly, embryos were removed and decapitated, and the brains were extracted from the skulls and placed in cold Hanks Balanced Salt Solution (HBSS). We dissected hippocampi of WT and *Lgdel* ^+/−^ embryos (C57/BL6J mouse background) and *dgcr8* WT and *Dgcr8* ^+/−^. We placed hippocampi in 0.125% trypsin-EDTA and incubated them for 30 minutes at 37 °C in a water bath to dissociate the tissue. We then blocked the trypsin activity by CNM, supplemented with 10% fetal bovine serum (FBS) and centrifuged the tubes for 5 minutes at 1200 rpm. The supernatant was discarded, and fresh CNM and 10% FBS were added. The hippocampi were dissociated gently by pipetting, and then the solution was filtered through a cell strainer and centrifuged for 10 minutes at 700 rpm. The supernatant was discarded, and cells were resuspended in CNM and counted using trypan blue and a hemocytometer. We seeded 100.000 cells/chip on the previously coated active area and then cultures were incubated at 37 °C with 5% CO_2_ and 95% humidity. After 1.5 hr, we added 1.6 ml of CNM to the culture chamber and incubated under the same conditions. On the other hand, we seeded 10.000 cells/well in BD Falcon 96-well imaging plates (Corning) for HCI experiments (BD Pathway 855, BD Biosciences). For maintenance and cell culture growth, one-third of the medium was routinely replaced with a fresh CNM every four days. All reagents were obtained, unless indicated differently, from Life Technologies.

### RNA extraction, cDNA preparation, and qRT-PCR

We extracted total RNA from WT, *Lgdel* ^+/−^, d*gcr8* WT, and *Dgcr8* ^+/−^ primary hippocampal cultures at 8, 16 and 26 DIVs (three embryos per condition) using QIAzol lysis reagent (Qiagen). Synthesis of cDNA from total RNA was performed with ImProm-IITM Reverse Transcription System (Promega). We performed qRT–PCR by using Quantifast SYBR Green method (Qiagen) with the following probes^11^; *NKCC1* (*Slc12a2*, NM_009194)-Fwd 5′-CCACCAGGAAACCATACCA-3′ and *NKCC1-Rev 5′*-AAGGCAGGCAAGTCTACC*-3′*, *KCC2* (*Slc12a5*, NM_020333)-Fwd 5′-GGACCACTAGCTGACCTC-3′ and *KCC2-Rev 5′*-CACCTGAGCCGTTTGATG-3′, *GAPDH* (NM_008084)-Fwd 5′-GAACATCATCCCTGCATCCA and *GAPDH*-Rev 5′-CCAGTGAGCTTCCCGTTCA-3′. Expression analysis was performed using the comparative cycle threshold (Ct) method.

### Immunofluorescence protocol

We performed immunostaining on mouse hippocampal cultures that were grown on CMOS-MEAs, and microplates using a previously described protocol^44^. Briefly, we removed the culture medium and washed using phosphate-buffered saline (PBS-1X) (Life Technologies) at 37 °C, and then we fixed samples in paraformaldehyde (4% in PBS-1X) for 15 minutes at room temperature (RT). We washed the sample 4x with PBS-1X, then cells were permeabilized with 0.1% Triton X-100 in PBS-1X (PBST) for 10 minutes, and then cells were blocked with 5% normal goat serum (NGS) (EuroClone) for 1 hr before incubation with primary antibodies. Cells were then incubated overnight at 4 °C in primary antibodies diluted in the NGS blocking buffer. The following primary antibodies were used: guinea pig anti-MAP-2 (Synaptic Systems; 1:1000), rabbit anti-c-fos (Calbiochem Millipore; 1:500), and rabbit anti-NKCC1 (Millipore; 1:100), and KCC2 (Millipore; 1:500). We performed three successive washing steps in PBST for five minutes per wash, and cells were incubated for 1 hr in the dark at RT with the corresponding secondary antibodies, including Alexa Fluor 488, Alexa Fluor 546, and Alexa Fluor 647 (all from Life Technologies; 1:1000). Subsequently, the cells were washed 4x with PBST and incubated for 15 minutes in the dark at RT with the nuclear marker Hoechst 33342 (Thermo Fisher Scientific; 1:500) diluted in PBS-1X. We visualized and acquired all images with 20X and 25X objective lenses using a BD Pathway 855 (BD Biosciences), and a Leica SP5 upright confocal microscope (Leica Microsystems), respectively.

### Drug treatments

#### Bicuculline methiodide

We prepared a fresh solution of bicuculline for each experiment by dissolving 8 mg/ml (2503-Tocris) in DDW. The final working concentration used for all experiments was 20 µM.

#### Bumetanide

We prepared a fresh solution of bumetanide for each experiment by dissolving 20 mg/ml (B3023-Sigma-Aldrich) in dimethyl sulfoxide (DMSO). The final working concentration used for all experiments was 10 µM.

### Extracellular recordings and analyses of spiking, bursting and dynamic changes

We performed multiple extracellular recordings from hippocampal neuronal networks grown on CMOS-MEAs at different developmental time points (DIVs). Each recording session lasted 10 minutes, and neural activity was acquired at a full-frame resolution of 7.8 kHz/electrode and from the whole array of 4096 electrodes (acquisition rate of 60 Mbyte/s). The time stamps of the spikes were detected using the precise timing spike detection (PTSD) algorithm of the Brainwave software application (3Brain GmbH). As in previous works, we adopted a threshold of 9x the standard deviation for spike detection, whereas burst events were identified if we detected at least five consecutive spikes in an inter-spike interval (ISI) lower than 100 ms. On the other hand, NBs were detected and analyzed as previously reported^69^. Only electrodes that recorded spikes with rates between 0.1 and 10 events/sec (active electrodes) were considered in our analysis. All spike trains were exported from BrainWave software in a MATLAB file format and were analyzed using custom Python scripts (Python Software Foundation). The analysis included first-order statistics of network-wide mean activity parameters (MFR and MBR), in addition to further analyses of the neuronal firing frequency distributions (lognormal-like distributions), NBs, and the distribution of IBIs. Lognormal-like distributions and fit statistics were analyzed as previously described^44^. Briefly, we assigned each active electrode to its corresponding averaged MFR value, then we normalized the occurrence of these active electrodes as a proportion of their corresponding spiking activity divided by the total number of electrodes in the array, which is 4096. For acceptance of NBs within a time-window of 150 msec (within a given temporal bin of 15 msec), the value of firing threshold was set approximately 15% of the total network spiking activity, and events were detected whenever the number of spiking activity exceeded this specific threshold. Finally, we quantified the distribution of IBIs as a bimodal temporal clustering of bursts that were binned using a Gaussian window with a 5% bin size of the burst duration, as previously reported^51^.

### Image analyses

To acquire images from neurons grown in 96 wells microplates, we used the BD Pathway 855 Bioimager in the non-confocal mode using 20x/0.75 NA objective (BD Biosciences) in the form of 3 × 3 montage (9 image fields/well).

#### c-fos

To quantify the neuronal activity through cellular c-fos expression, we grew neuronal cultures on two different cell culture platforms. First, neuronal networks grown on CMOS-MEAs were acquired on a Leica SP5 upright confocal microscope using a 25x/0.95 NA objective (Leica Microsystems). Second, to consolidate the cellular readouts of c-fos expression in high-throughput experimentation, we performed multiplexed HCI by growing neurons in 96 wells microplates (Corning). Consequently, the quantification of c-fos expression for all these data was performed by counting the c-fos-positive nuclei. Images acquired from CMOS-MEAs were quantified using the object count tool of the NIS-Element Advance Research Software AR (Nikon), which allowed us to perform automated object detection and counting by using image thresholding based on area, circularity, and EqDiameter parameters (Nikon NIS-Elements Advanced Research User’s Guide V.4). Images were acquired from at least nine fields in each confocal image from four different preparations and different animals. For data acquired with the HCI platform, we quantified the c-fos-positive nuclei ratio using BD AttoVision software v1.6 (BD Biosciences), which allowed us to apply the polygon segmentation methods to generate polygon-shaped ROIs from a single dye (c-fos and Hoechst for the nuclei); then, the total number of ROIs indicated the positive expression of c-fos with respect to their nuclei.

#### NKCC1 and KCC2

We quantified the average intensity expressions of the chloride cotransporters to investigate the GABAergic-signaling switch in *Lgdel* ^+/−^ and *dgcr8* ^+/−^. Neurons from WT, *Lgdel* ^+/−^, and *dgcr8* ^+/−^ embryos were grown in 96 wells microplates (Corning). We measured the expression intensity of NKCC1 and KCC2 by using multiplexed immunofluorescence HCI. The level of chloride cotransporters was analyzed using BD AttoVision software v1.6 (BD Bioscience) by quantifying the gray level in the segmented polygon-shaped ROIs. Given that NKCC1 is a nonspecific neuronal protein, we performed a segmentation method based on two dyes (channel A) for NKCC1, and (channel B) for MAP-2, thus resulting in two output ROIs (one for each channel). The sum of the averaged intensity in ROIs from NKCC1 (channel A) was selected for quantification only if they matched the segmented ROIs of the neuronal MAP-2 expression (channel B). Under this condition, the averaged intensity in ROIs from NKCC1 was considered as the final expression of NKCC1 at its associated time-point. On the other hand, KCC2 protein is a neuronal-specific marker, thus the polygon segmentation methods were used to generate polygon shaped ROIs from a single dye (nucleus), and the sum of the averaged intensity values of these individual ROIs was computed as the final expression of KCC2 at a precisely measured time-point.

#### Neurite Outgrowth

We performed morphometric measurements of neurite processes on WT, *Lgdel* ^+/−^ and *Dgcr8* ^+/−^ mouse embryonic hippocampal cultures at 5 DIVs grown in 96 wells microplates. The images were analyzed using BD’s neurite outgrowth algorithms (BD Bioscience). The neurite outgrowth analysis required two dye channels; (channel A) for the nucleus and (channel B) for the neurites of a neuron. Therefore, the Hoechst (channel A) was used to generate segmentation masks for the nuclei and to create cell body boundaries around them. Next, MAP-2 (channel B) was used to define the structure of the neuronal network. After analysis, image masks were generated as illustrated in (Fig. 7a). Finally, neurite outgrowth parameters (Table 2, see also BD AttoVision User’s Guide V1.6), including neurite total and maximum length per cell, the number of roots/neurites emerging per cell, neurite extremities (primary, secondary, and tertiary branching if exist), were generated and exported as text files for further quantifications.

### Statistical analyses and experimental parameters

All experimental data reported in this work are expressed as the mean ± standard error of the mean (SEM). Differences between groups were examined for statistical significance, where appropriate using one-way analysis of variance (ANOVA) or two-way ANOVA followed by Tukey’s posthoc testing. A non-parametric Kruskal-Wallis method was used for data that were not normally distributed. A non-parametric Kolmogorov-Smirnov test was used to assess differences between distributions and *p* < *0.05* was considered significant. The statistical tests used for each specific experiment are depicted in the Figures’ legends.

To ensure the consistency and reproducibility of our results, we conducted at least three repeated trials for each experimental condition. These tests were performed in different cell culture preparations and from at least three different animals. The number of samples used in each experiment is depicted in the Figures’ legends.

## Electronic supplementary material

Supplementary Information

Supplementary movie a

Supplementary movie b

Supplementary movie c
