# Supplementary material for: Developmental excitatory-to-inhibitory GABA-polarity switch is disrupted in 22q11.2 deletion syndrome: a potential target for clinical therapeutics
Source: Sci Rep. 2017 Nov 16;7:15752. doi: 10.1038/s41598-017-15793-9 (PMC5691208; doi:10.1038/s41598-017-15793-9)
Supplement: Supplementary file 1 — Supplementary Information [file 41598_2017_15793_MOESM1_ESM.pdf]

Developmental excitatory-to-inhibitory GABA-polarity switch is disrupted in 22q11.2 deletion syndrome: a potential target for clinical therapeutics

Hayder Amin*┼[1], Federica Marinaro┼[2,3], Davide De Pietri Tonelli[2§], and Luca Berdondini[1§]

[1]Nets[3] Laboratory, Departement of Neuroscience and Brain Technologies (NBT), Fondazione Istituto Italiano di Tecnologia (IIT), Via Morego 30, 16163 Genoa, Italy.
[2]Neurobiology of miRNA Laboratory, Department of Neuroscience and Brain Technologies (NBT), Fondazione Istituto Italiano di Tecnologia (IIT), Via Morego 30, 16163 Genoa, Italy.
[3]currently at the Gurdon Institute and Dept. of Biochemistry, University of Cambridge, Tennis court road, Cambridge CB2 1QN, UK.

*Correspondence to hayder.amin@iit.it
┼ equal contributions
§ co-last

SUPPLEMENTARY FIGURES, LEGENDS, TABLES, AND MOVIES

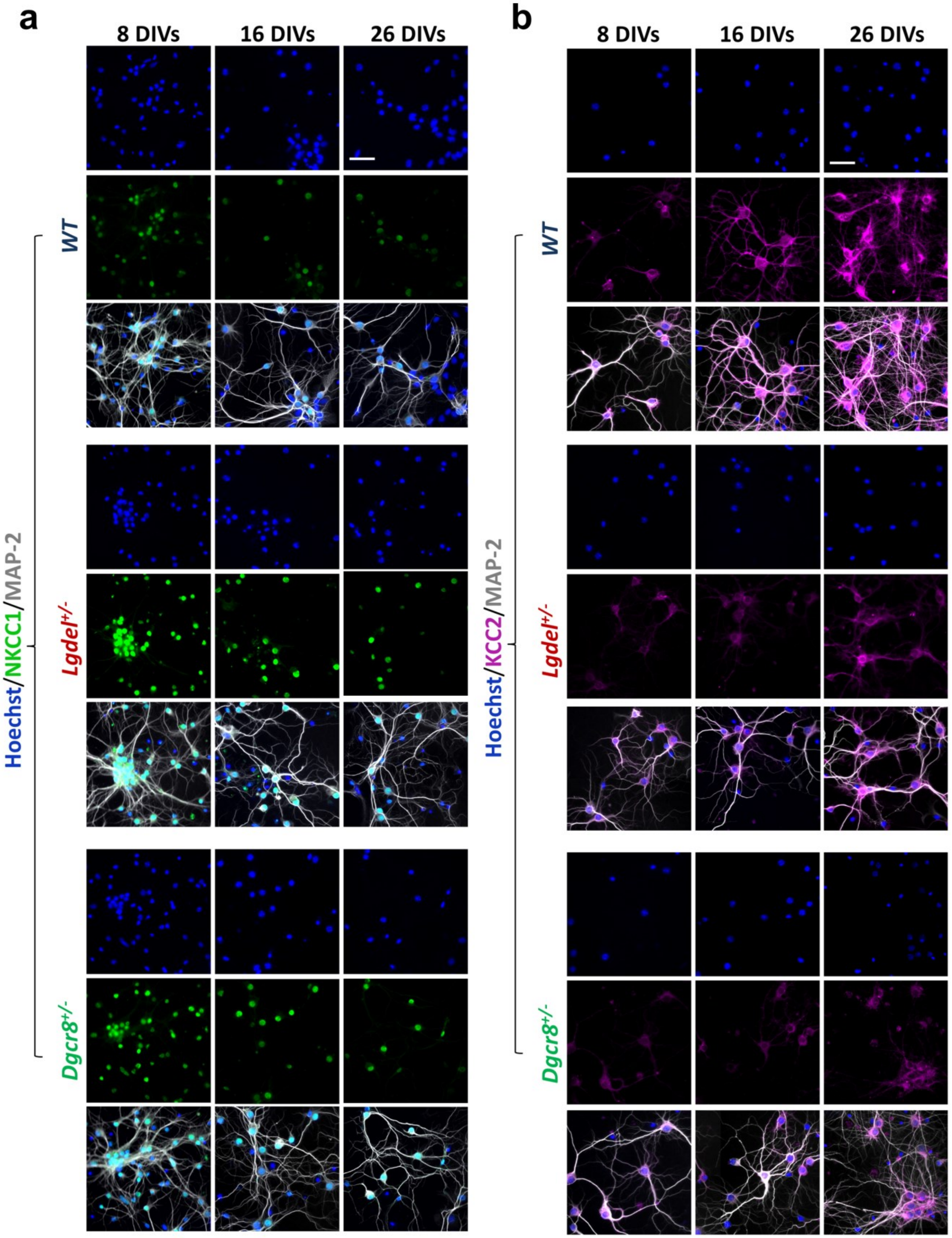
a
b
8 DIVs
16 DIVs
26 DIVs
8 DIVs
16 DIVs
26 DIVs
WT
Lgdel+/-
Dgcr8+/-
Hoechst/NKCC1/MAP-2
WT
Lgdel+/-
Dgcr8+/-
Hoechst/KCC2/MAP-2

Figure S1 | Abnormal expression of NKCC1 and KCC2 in *Lgdel*$^{+/-}$ and *Dgcr8*$^{+/-}$ neurons. Extended information from Fig. 2a,c.

(a) Fluorescence micrographs showing the changes in intensity expression of NKCC1 in the three animal genotypes at 8, 16, 26 DIVs. Scale bar represents 30 μm. **(b)** As in (a), but for changes in intensity expression of KCC2. Scale bar represents 30 μm.

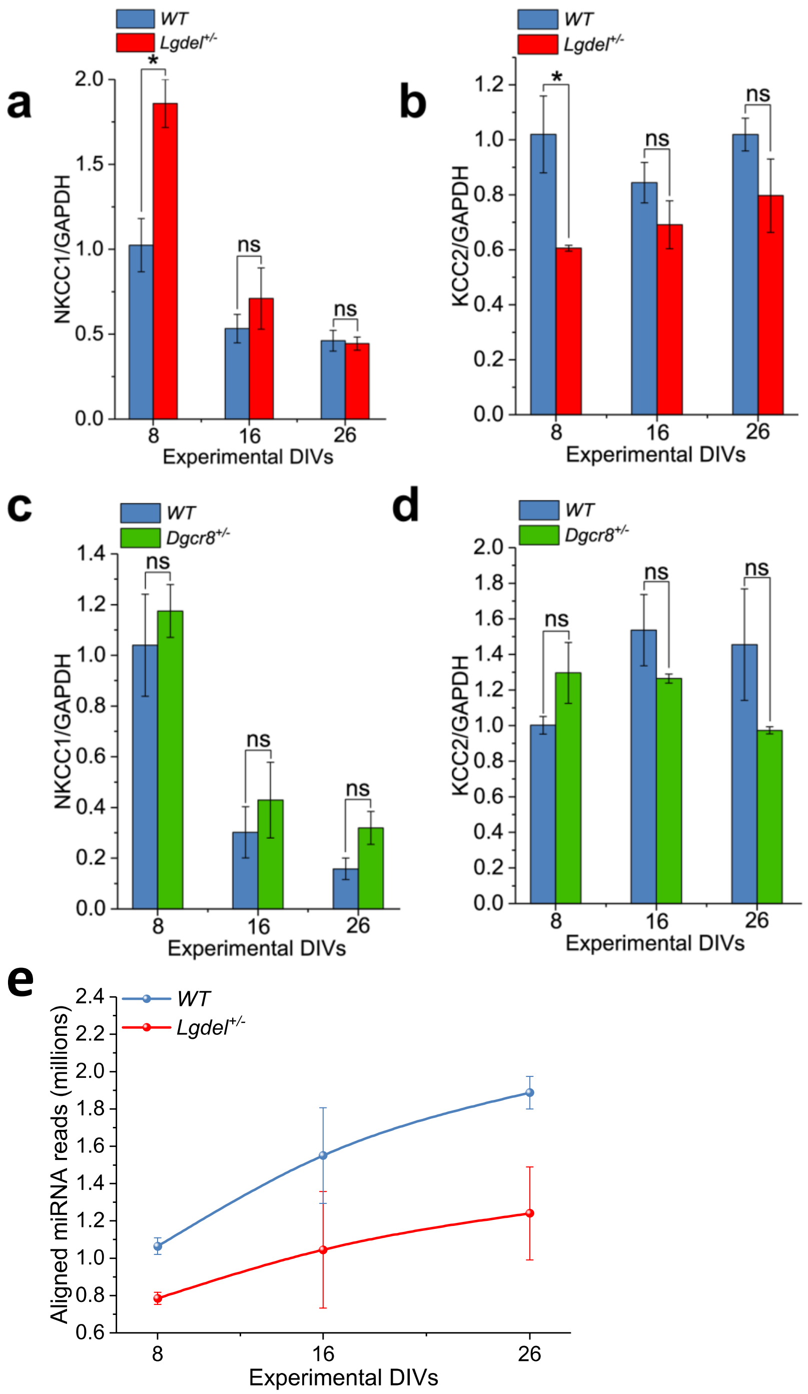

Figure S2 | Molecular quantifications of NKCC1 and KCC2 transcript and miRNA expression level in WT, *Lgdel*$^{+/-}$ and *Dgcr8*$^{+/-}$ cultures.

(a) The transcript expression of NKCC1 in *Lgdel*$^{+/-}$ cultures, as assayed by qRT-PCR, shows at 8 DIVs a significantly higher level compared to WT (*$p < 0.05$, ANOVA), but non-significant changes at 16 and 26 DIVs. **(b)** As in (a), but for KCC2 transcript in *Lgdel*$^{+/-}$ cultures. **(c)** As in (a), but for *Dgcr8*$^{+/-}$ cultures showing no significant changes in the transcript expression compared to WT at 8, 16, and 26 DIVs. **(d)** As in (b), but for *Dgcr8*$^{+/-}$ cultures. **(e)** The read counts for all genome-aligned miRNA sequences identified in WT and *Lgdel*$^{+/-}$ hippocampal neuronal cultures at 8, 16, and 26 DIVs. The miRNA reads are significantly lower in *Lgdel*$^{+/-}$ cultures compared WT, all over the developmental stages ($p < 0.05$, ANOVA).

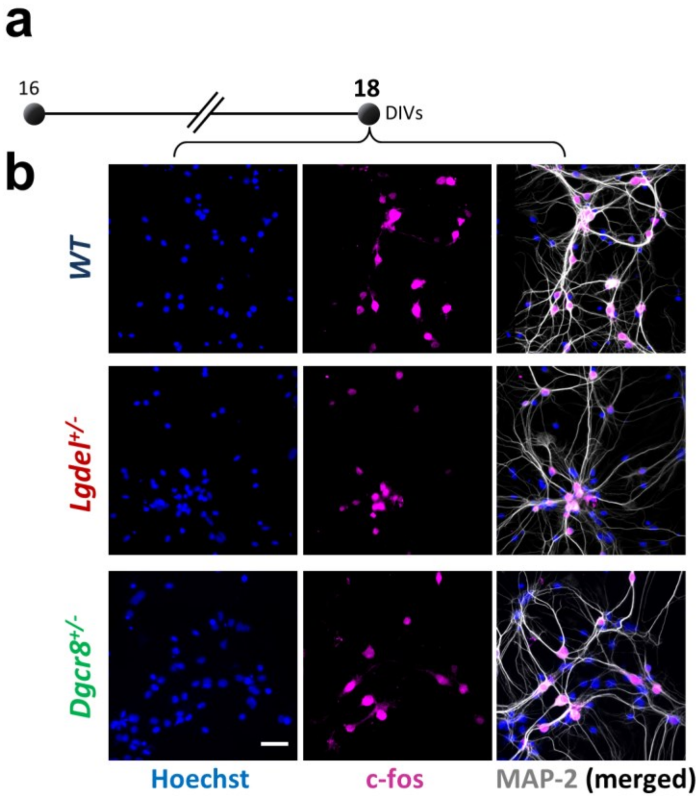

Figure S3 | Optical readouts of c-fos cellular activity in WT, *Lgdel*$^{+/-}$ and *Dgcr8*$^{+/-}$ neurons. Extended information from Fig. g-i.

(a) Schematic summary of the experimental implementation to assay at 18 DIVs under the untreated condition (without bicuculline) the cellular neuronal activity changes in the three animal genotypes. **(b)** Fluorescence micrographs showing the c-fos expression in the untreated neuronal cultures. Scale bar represents 30 µm.

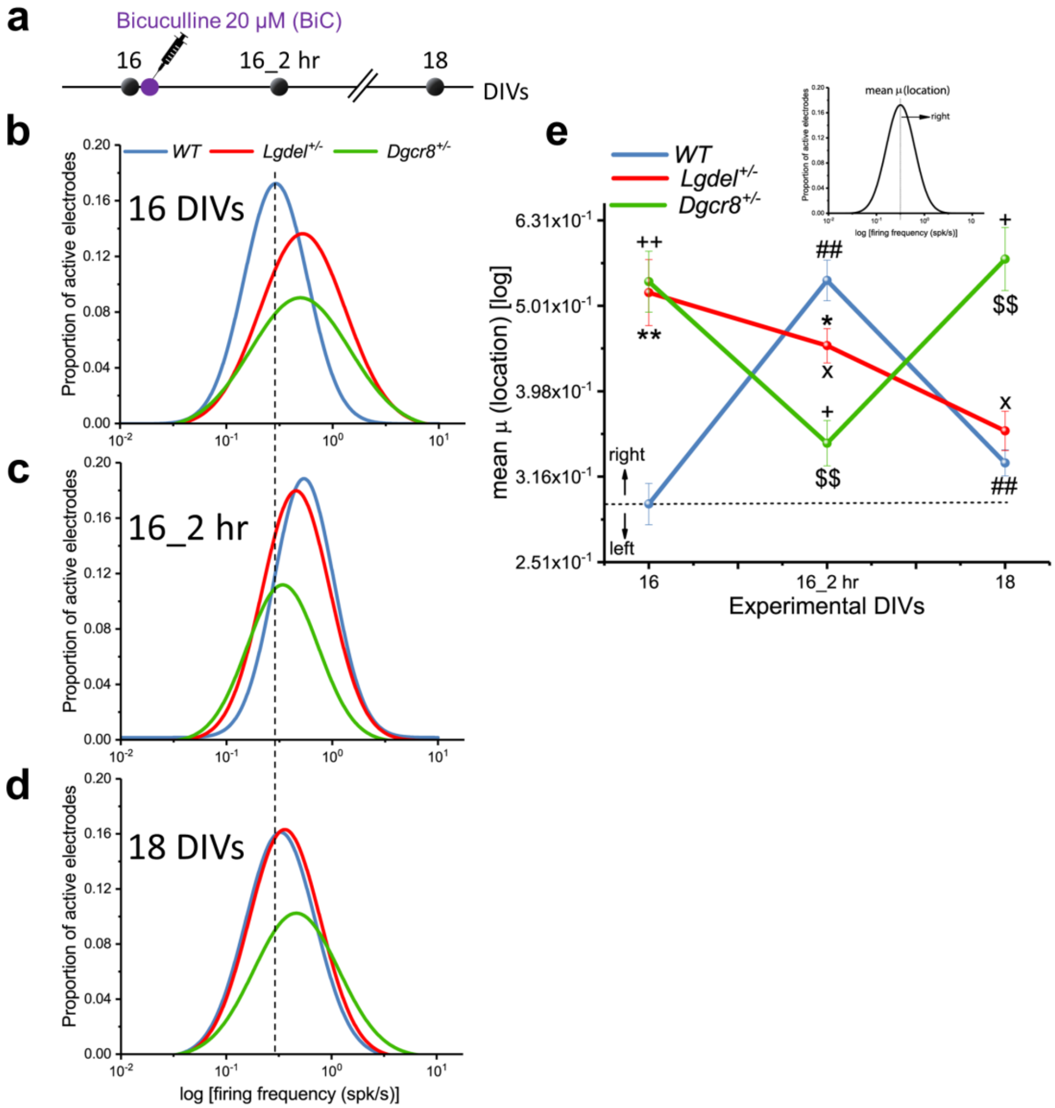

Figure S4 | Changes in network dynamics induced by dysregulated homeostatic plasticity in *Lgdel*$^{+/-}$ and *Dgcr8*$^{+/-}$ neuronal networks.

(a) Schematic summary of the experimental protocol to test the homeostatic plasticity responses to increase network firing by 20 μM bicuculline. **(b)** Lognormal-like distributions of firing rates (WT, *Lgdel*$^{+/-}$ and *Dgcr8*$^{+/-}$ networks) at 16 DIVs before bicuculline-treatment, showing an already significant shift toward higher frequencies (*right*) of *Lgdel*$^{+/-}$ and *Dgcr8*$^{+/-}$ networks compared to WT ($p < 0.05$, Kolmogorov-Smirnov test). **(c)** Same as in (b), but 2 hr after bicuculline-treatment, lognormal-like distribution of WT network, showing a significant shift to (*right*) compared to WT network before bicuculline-treatment, while distributions of *Lgdel*$^{+/-}$ and *Dgcr8*$^{+/-}$ networks shift significantly to (*left*) ($p < 0.05$, Kolmogorov-Smirnov test). **(d)** Same as in (b) and (c), but 48 hr after bicuculline-treatment, distributions of WT and *Lgdel*$^{+/-}$ networks shift to (left) compared to their previous condition (16_2 hr), while *Dgcr8*$^{+/-}$ networks shift significantly to (right). **(e)** Quantification of the peak location of the lognormal-like distribution indicated by the mean μ shows the dynamical changes before-and-after bicuculline-treatment in WT, *Lgdel*$^{+/-}$ and *Dgcr8*$^{+/-}$ networks (significant shifts toward high and low firing frequencies) (** denotes $p < 0.01$ *Lgdel*$^{+/-}$ vs. WT, ++ denotes $p < 0.01$ *Dgcr8*$^{+/-}$ vs. WT, ## denotes $p < 0.01$ WT vs. WT, x denotes $p < 0.05$ *Lgdel*$^{+/-}$ vs. *Lgdel*$^{+/-}$, $$ denotes $p < 0.01$ *Dgcr8*$^{+/-}$ vs. *Dgcr8*$^{+/-}$, ANOVA). The *top right* inset illustrates a prototype of a firing distribution on a logarithmic scale when its mean μ shifts to the (right) toward high firing frequencies. Dashed lines indicate the peak and the location of the Gaussian distribution of the WT pertained to 16 DIVs.

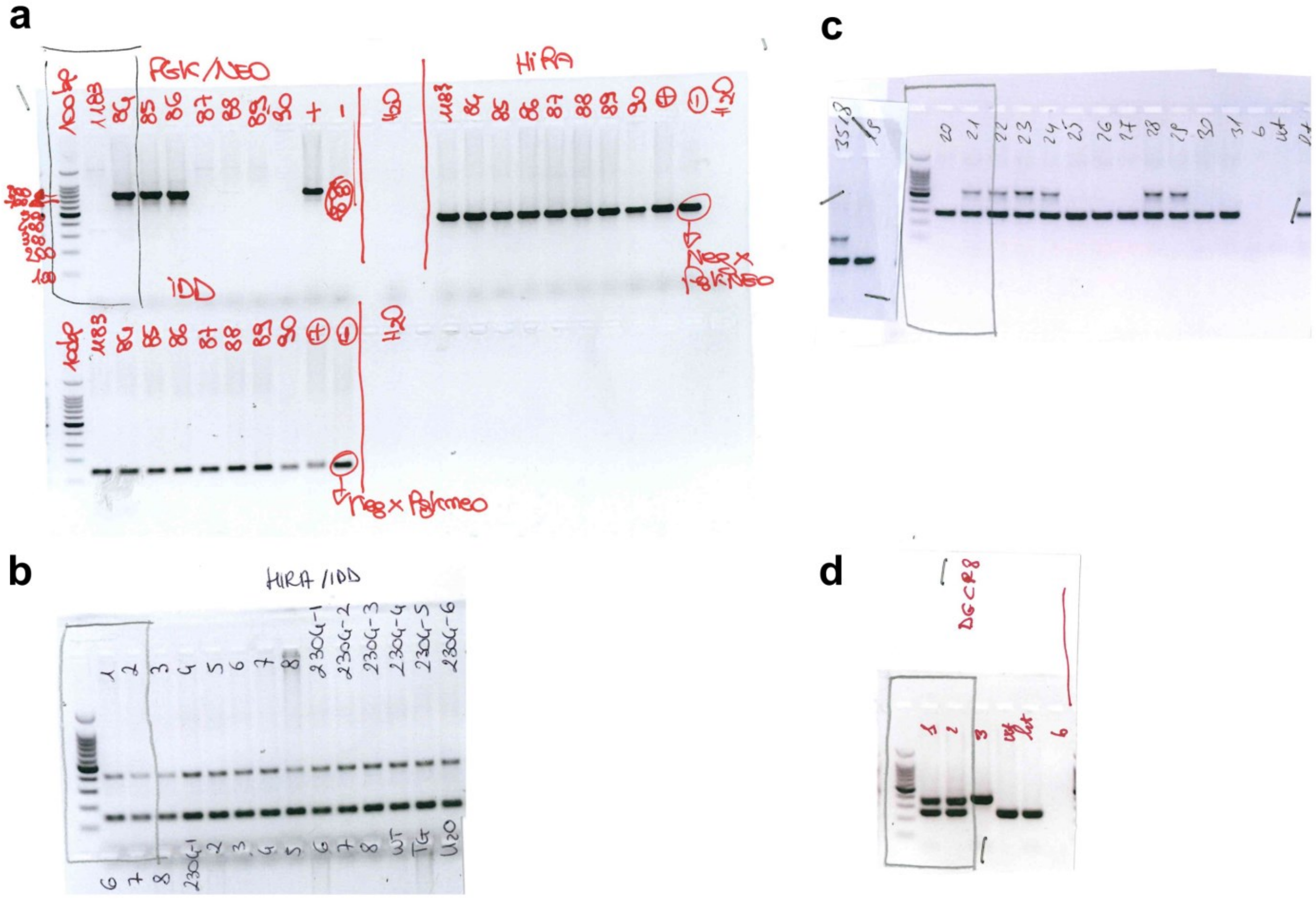

Figure S5 | Full-length agarose gel images. Extended data from Figure 1a.

(a) and **(b)** Groups of animals genotyped for *PGK/Neo* (*top*), and *Idd/Hira* alleles (*bottom*) (WT and *Lgdel*$^{+/-}$ embryos). **(c)** and **(d)** Groups of animals genotyped for *Cre* (top), and *flox* alleles (bottom) (Dgcr8 WT and *Dgcr8*$^{+/-}$ embryos). The free-hand rectangles correspond to the cropped regions of gels in Figure 1a.

Table 1 | List of primers used in this study for genotyping

Primer	Primer Sequence	Product size (bp)	Annealing T	Mouse Model
Idd wt Fwd<br>*Idd* wt Rev	5’-CTGTTGTTGACACAGCACATG-3’<br>5’-AACTCTACCTGTTCCTACTG-3’	WT 500<br>*Lgdel*$^{+/-}$ 500	58°C	*Lgdel*$^{+/-}$
Hira Fwd<br>*Hira* Rev	5’-TCTTGCAACTCTGAGAGGTC-3’<br>5’-GTGATGCTAGTCTCTAGCTG-3’	WT 150<br>*Lgdel*$^{+/-}$ 150	58°C	*Lgdel*$^{+/-}$
PGK1 Fwd<br>*Neo5F* Rev	5’-GCTAAAGCGCATGCTCCAGAC-3’<br>5’-ACCGCTATCAGGACATAGCGT-3’	*Lgdel*$^{+/-}$ 800	58°C	*Lgdel*$^{+/-}$
Dgcr8 Fwd<br>*Dgcr8* Rev	5’-GACATCAATCTGAGTAGAGACAGG-3’<br>5’-CAGATGGTAACTAACCTGCCAACC-3’	WT 244<br>flox 370	60°C	*Dgcr8*$^{+/-}$
Emx1 Fwd<br>*Emx1* Rev<br>*Emx1* Cre	5'-GTGAGTGCATGTGCCAGGCTTG-3'<br>5'-TGGGGTGAGGATAGTTGAGCGC-3'<br>5'-GCGGCATAACCAGTGAAACAGC-3'	wt 200<br>cre 500	65°C	*Emx1*

Table 2 | Neurite outgrowth measurements

Neurite Outgrowth Measurement	Function/Description
Neurite total length	Total length (pixels) of all segments of each cell body.
Neurite maximum length	Maximum length (pixels) of the longest neurite segment for each cell body.
Neurite extremity counts	Total number of primary, secondary, tertiary, etc., extremities per cell body.
Neurite root counts	Total number of roots from the cell body (number of separate neurite trees).
Neurite segment count	Total number of segments between node points per cell body. Node points can be from secondary, tertiary, etc., branching or intersection with segments from other cell bodies.
Neurite node points	Total number of points where secondary, tertiary, etc., branches exist per cell body.

Supplementary movies | Samples of spontaneous firing activity from WT, *Lgdel*$^{+/-}$ and *Dgcr8*$^{+/-}$ neuronal networks.

(a) Large-scale electrical recording from WT neuronal network at 8 DIVs illustrates the synchronous firing activity within the network indicated by three selected active electrodes (within 100 ms of activity duration). **(b)** Same as in (a), but from *Lgdel*$^{+/-}$ neuronal network showing the asynchronous activity among active electrodes. **(c)** Same as in (a), and (b), but from *Dgcr8*$^{+/-}$ neuronal networks displaying the synchronous firing activity.
